# Shot-location polarization and post-2018 pace stabilization in the NBA: Evidence from 21 seasons of team and shot-coordinate data (2004–2025)

**DOI:** 10.1371/journal.pone.0358360

**Published:** 2026-09-18

**Authors:** Shuming Cui, Zhenxing Yang, Hemu Du, Rongheng Liu, Shijie Liu

**Affiliations:** 1 School of Physical Education, Shanghai University of Sport, Shanghai, China; 2 Department of Physical Education, Capital Normal University, Beijing, China; 3 College of Teacher Education, Capital Normal University, Beijing, China; Universidade Federal de Goias, BRAZIL

## Abstract

NBA offenses increasingly reflect a “space-and-pace” approach, yet it remains unclear whether recent tempo changes primarily reflect long-run strategic adaptation or the 2018–19 14-second offensive-rebound shot-clock reset. We combined team-season metrics with shot-location data from the NBA Stats API across 21 seasons (2004–05–2024–25; > 420,000 field-goal attempts). Analyses included spatial heatmaps and a Spatial Allocation Index (SAI), segmented regression for league Pace, and multivariate OLS models for Offensive Rating (ORtg). Heatmaps showed a substantial reduction in mid-range attempts and greater concentration at the rim and beyond the arc; SAI increased from 58.98% to 69.99%. Segmented regression identified a marked change around 2018–19, with an immediate increase in Pace and a post-2018 trend that flattened and became slightly negative (pre-2018 slope ≈ 0.44 possessions/season; post-2018 net slope ≈ −0.26). Turnover percentage declined despite higher tempo (p < 0.001). The 2018–19 reset aligned with a step change in tempo, while subsequent seasons show consolidation rather than continued acceleration. Over the longer run, shot allocation became increasingly concentrated at the rim and from three-point range, indicating co-evolution in tempo and shot-location strategy.

## 1. Introduction

### 1.1. The evolution paradigm

The NBA has shifted from a slower, position-based half-court style to today’s “space-and-pace” approach [1]. This shift has occurred alongside wider use of game data and tracking tools. Research notes that optical tracking systems (e.g., SportVU) allow teams to evaluate possessions with quantitative indicators rather than relying mainly on experience [2]. In this context, Expected Possession Value (EPV) is often used to link on-court actions with expected outcomes. This development also made the spatial distribution of shots increasingly measurable, enabling analysts to quantify where shots were taken and how shooting profiles varied across court locations [3].

This direction is consistent with previous stochastic modeling work [4]. Their results suggest that offensive outcomes depend strongly on spatial choices, especially creating attempts in areas that tend to be more valuable. From this viewpoint, the long-term move toward the rim and the three-point line can be described as a change in shot allocation toward high-value regions, rather than simply a stylistic trend.

To describe the evolution clearly, offensive output should be separated from game speed. Research points out that per-game statistics can be misleading because they are mechanically affected by pace [5]. Possession-based metrics allow cleaner comparisons across seasons. The shift is also visible at the possession level. A comparison of the 1990s Chicago Bulls with the recent Golden State Warriors reported shorter possession durations in the modern era [6]. Shorter time-to-shot is consistent with faster decision cycles and earlier shot attempts. At the player level, physiological state has also been associated with shooting accuracy, suggesting that faster offensive environments may interact with execution-related constraints [7,8].

Finally, the change appears to have begun before the most recent rule adjustments. Using league data from 2000 to 2017, research reported steady increases in pace and three-point volume, alongside a decline in mid-range attempts [9] Together, these trends suggest that “space and pace” mainly reflects gradual strategic learning about shot-location value, rather than being explained only by external rule changes.

### 1.2. The “efficiency paradox”

In motor-control research, the speed–accuracy trade-off describes the general tendency for increased movement speed or greater temporal demands to constrain movement accuracy [10]. Basketball-specific evidence also indicates that execution accuracy can vary with exercise intensity; for example, free-throw accuracy has been shown to change across different exercise-intensity conditions in young basketball players [11]. Applied cautiously at the team level, these findings raise the possibility that faster play could increase execution demands and potentially contribute to possession errors. However, recent NBA trends do not fully follow this expectation. In this study, we use the term “efficiency paradox” to describe the pattern that tempo increased while turnover rates did not increase in parallel.

Evidence from prior work points in the same direction. Research reported a long-run rise in pace alongside changes that were consistent with shifts in shot selection rather than a move toward more chaotic play [9]. Other studies have also found that higher-performing teams can sustain strong offensive output while keeping turnovers low, even in faster games [12]. Taken together, these findings suggest that the link between speed and error may be weaker in the modern NBA than the traditional account would imply. What remains less clear is why this pattern occurs. One possibility is that changes in spacing and offensive structure alter the spatial constraints under which offensive decisions are made. Previous tracking-based research has demonstrated that defensive effectiveness and player interactions have a measurable spatial structure [13]. Whether such spatial changes make decision-making more stable at higher tempo, however, requires more direct testing.

### 1.3. The 2018 rule intervention

Strategic adaptation within the league—most notably the growth in three-point volume—likely explains much of the NBA’s offensive shift. At the same time, rule changes may have contributed to how quickly tempo increased. Before the 2018–19 season, the NBA adjusted the shot clock after an offensive rebound: instead of resetting to 24 seconds, it resets to 14 seconds [14].

The stated aim was to increase possessions and reduce slow second-chance sequences. The shorter reset also reduces the time available for re-organization after an offensive rebound, which may increase time pressure on decision-making and execution. Research notes that basketball already involves repeated high-intensity actions and substantial metabolic demand [15]; shorter decision windows may therefore add constraints, especially across long stretches of play. In this context, it is useful to examine whether the pace trend changed around 2018–19, and whether the post-2018 pattern is more consistent with continued acceleration or with a flattening of tempo growth under practical constraints.

It should also be noted that earlier officiating changes affected the defensive environment. In particular, the NBA tightened restrictions on hand-checking against ball handlers in 2004, reducing the degree of permissible on-ball contact [16]. These changes predate the primary breakpoint examined here and are therefore treated as part of the broader baseline conditions under which subsequent strategic adaptation occurred.

### 1.4. Study objectives

To distinguish long-run strategic change from the 2018–19 rule adjustment, this study analyzes a spatiotemporal dataset covering 21 NBA seasons (2004–05–2024–25). Following common approaches in basketball analytics [5], we pursue three objectives:

Quantify spatial evolution. We construct a Spatial Allocation Index (SAI) to summarize how shot attempts shifted away from mid-range areas and toward the rim and the three-point line. SAI captures shot-location allocation rather than shooting efficiency or expected value, and is used here to describe long-run changes in league-wide shot distribution [1].

Assess tempo changes around 2018–19. We apply segmented regression to examine whether the Pace time series shows a change in level or trend around the 2018–19 shot-clock reset, and whether the post-intervention trend is consistent with a flattening of tempo growth.

Examine correlates of offensive output. Using multivariate models, we evaluate associations between Offensive Rating (ORtg) and key indicators such as pace, three-point attempt rate, and offensive rebounding, to assess whether higher offensive output in the modern era aligns more closely with tempo, shot allocation, or possession extension [17].

## 2. Methodology

### 2.1. Data acquisition and preprocessing

We assembled a dataset covering 21 NBA regular seasons (2004–05–2024–25). Two complementary data sources were combined to capture both long-term league trends and fine-grained shot-location patterns.

Team-season data (macro level). Team performance indicators, including win–loss records, offensive rating (ORtg), pace, turnover percentage (TOV%), offensive rebound percentage (ORB%), and shooting-efficiency metrics, were obtained from Basketball-Reference.com. These data were used to characterize season-level changes in offensive production, game tempo, and possession-related performance.

Shot-location data (micro level). Shot-location information was retrieved from publicly accessible NBA Stats endpoints using the Python package nba_api (version 1.11.3). Specifically, shot-level records were collected through the ShotChartDetail endpoint, which provides Cartesian court coordinates (LOC_X, LOC_Y) for individual field-goal attempts. Only regular-season games were included in the analysis.

The public NBA Stats API does not provide detailed documentation regarding the underlying coordinate acquisition procedures or measurement uncertainty across seasons. Consequently, shot coordinates were treated as archival event-location records, and the same spatial classification thresholds were applied consistently throughout all seasons.

Because classification was rule-based and deterministic, repeated application of the same coordinate thresholds to an identical record produced the same spatial category; however, external validation against an independent coordinate source was not available.

Data preprocessing. Retrieved shot records were screened for completeness and consistency before analysis. Shot records with invalid court coordinates outside the standard NBA half-court plotting range were excluded. The remaining records were used to generate shot-density visualizations and season-level spatial indicators.

Following preprocessing, each field-goal attempt was assigned to a spatial category based on its recorded location. These classified shot records were subsequently used to construct season-level spatial indicators and generate shot-density visualizations.

### 2.2. Variable definitions

To compare seasons with different game speeds, we expressed key indicators on a possession basis rather than per game, consistent with common practice in basketball analytics (Kubatko et al., 2007) [5,18].

Pace. Estimated possessions per 48 minutes. Pace is used here as a summary indicator of game tempo, reflecting how frequently offensive possessions occur.

Offensive Rating (ORtg). Points scored per 100 possessions. ORtg provides a pace-adjusted indicator of offensive output by separating scoring productivity from the number of possessions.

Point Attempt Rate (3PAr). Three-point attempts as a share of total field-goal attempts. 3PAr is included as a descriptive indicator of perimeter-oriented shot selection and complements the broader spatial-allocation measure represented by SAI.

Turnover Percentage (TOV%). Turnovers per possession. Unlike raw turnover counts, TOV% captures possession security on a normalized scale, which is important when pace differs across seasons.

Offensive Rebound Percentage (ORB%). Offensive rebounds divided by available offensive rebound opportunities. ORB% is used to represent second-chance possession generation.

True Shooting Percentage (TS%). A shooting efficiency measure that accounts for field goals and free throws. TS% is included as a pace-neutral indicator of scoring efficiency.

Rationale for metric selection. Variable selection was guided by prior work on key determinants of basketball outcomes and by the broader use of performance indicators in sport-performance analysis [19]. Dean Oliver’s “Four Factors” were evaluated using directed acyclic graphs (DAGs), highlighting the central role of shooting efficiency and the importance of turnover control [20]. In this study, we therefore focus on indicators that reflect shot selection, scoring output, and possession security across seasons.

### 2.3. Mathematical definitions and statistical models

Next, we connect the spatial patterns derived from shot coordinates to statistical models. This step matters because box-score summaries do not describe where shots are taken or how shot geography changes across seasons. Previous basketball analytics research has noted that many traditional indicators miss the context-sensitive nature of basketball possessions [21]. Here, we use shot-location data to summarize long-run changes in spacing and shot distribution, and then combine these summaries with time-series and regression models to examine how these changes co-move with league-wide tempo and offensive output. Importantly, the models are used to describe associations and temporal alignment rather than to make strong causal claims about the effects of a single rule change.

#### 2.3.1. Spatial Allocation Index (SAI).

To summarize league-wide shifts in shot distribution, we construct a Spatial Allocation Index (SAI). The motivation comes from spatial analytics work that uses location information to describe how teams distribute shot attempts across the court [22]. However, unlike approaches that combine location with points per shot, SAI is intentionally defined as an allocation metric: it captures where shots are taken, not whether those shots are made.

SAI measures the share of field-goal attempts taken in two high-value regions in modern NBA shot selection—the restricted area and the three-point zone—relative to total attempts:


$$\begin{array}{c} SAI=\frac{FGA_{Rim}+FGA_{\text{3}PT}}{FGA_{Total}}\times \text{100}\% \end{array}$$ (1)

where FGA_Rim_ denotes attempts classified as restricted area shots according to the NBA shot-location taxonomy, FGA_3PT_ denotes attempts beyond the three-point line, and FGA_Total_ is total field-goal attempts in a season.

Because SAI is scaled by total attempts, it is a composition measure rather than a volume measure. This reduces sensitivity to season-to-season differences in overall tempo and allows cleaner comparisons of shot-allocation tendencies. SAI should be interpreted as a structural indicator of shot-location preference, complementary to outcome-based measures such as eFG%, TS%, or ORtg. In other words, a higher SAI indicates greater concentration of attempts at the rim and beyond the arc, but it does not, by itself, imply higher shooting efficiency.

The rationale for SAI is that modern NBA offenses increasingly concentrate shot attempts in two locations that are generally associated with favorable expected scoring outcomes: the restricted area and the three-point zone. By combining the relative frequencies of attempts from these two regions, SAI provides a parsimonious summary of league-wide shot-allocation tendencies while remaining independent of shooting accuracy. Consequently, SAI reflects offensive spatial preferences rather than offensive effectiveness itself.

Unlike three-point attempt rate (3PAr), which captures only one component of modern shot selection, SAI incorporates both rim and perimeter shot allocation and therefore provides a more comprehensive description of spatial shot concentration across seasons.

To evaluate the associations between SAI and offensive-performance indicators while accounting for repeated observations from the same NBA franchises, separate linear mixed-effects models were fitted for ORtg, TS%, eFG%, and winning percentage, with SAI entered as a fixed effect and franchise included as a random intercept. Pearson correlation coefficients were retained as descriptive measures only. Model fit for the linear mixed-effects models was summarized using marginal R^2^, representing variance explained by the fixed effects, and conditional R^2^, representing variance explained jointly by the fixed and random effects.

#### 2.3.2. Segmented regression model.

To examine whether the league-average Pace series shows a change around the 2018–19 shot-clock reset (14 seconds after an offensive rebound), we use segmented regression in an interrupted time-series (ITS) framework, following established methodological recommendations for evaluating population-level interventions [23].

This approach estimates (i) an immediate level change and (ii) a change in the post-2018 trend in Pace. The 2018–19 season was selected a priori because it corresponds to the implementation of the NBA offensive-rebound shot-clock reset rule, which reduced the reset duration from 24 to 14 seconds.


$$\begin{array}{c} Pace_{t}=\beta_{\text{0}}+\beta_{\text{1}}\cdot t+\beta_{\text{2}}\cdot D_{t}+\beta_{\text{3}}\cdot (t-T^{*})\cdot D_{t}+\in_{t} \end{array}$$ (2)

where Pace_t_ is league-average Pace in season t; t is the season index (centered at 2005); T^*^is the breakpoint year (set to 2018); and Dt equals 1 for post-2018 seasons and 0 otherwise. β_1_ captures the pre-2018 trend, β_2_ captures the immediate level change around 2018–19, and β_3_ captures the change in slope after 2018.

Because the analysis uses a single league-level series with one intervention point, the ITS results are interpreted as temporal alignment and trend change, not as definitive causal estimates of the rule’s isolated effect. To account for potential serial correlation and heteroskedasticity in the league-level time series, segmented-regression coefficients were estimated using HAC/Newey–West standard errors with a maximum lag of 2. Given the limited number of seasonal observations, test statistics were calculated by dividing each coefficient estimate by its HAC standard error, and two-sided p-values and 95% confidence intervals were based on a t distribution with 17 residual degrees of freedom. Model fit was summarized using R^2^ and adjusted R^2^, and residual autocorrelation was assessed using the Durbin–Watson statistic. Because the analysis included the full set of 21 available NBA seasons rather than a prospectively sampled cohort, an a priori power calculation was not applicable. As a sensitivity analysis, minimum detectable effects were calculated for 80% power at a two-sided α level of 0.05 using the model-specific HAC standard errors and 17 residual degrees of freedom.

#### 2.3.3. Factors associated with offensive output.

To examine factors associated with offensive output, we estimate a multivariate ordinary least squares (OLS) model with Offensive Rating (ORtg) as the dependent variable. Predictors include (i) tempo (Pace), (ii) shot-location strategy (3PAr), and (iii) second-chance creation (ORB%). By including these variables jointly, we assess whether variation in ORtg is more closely aligned with tempo, shot mix, or possession extension.


$$\begin{array}{c} \text{ORtg}=\alpha +\beta_{\text{1}}\cdot \text{Pace}+\beta_{\text{2}}\cdot \text{3PAr}+\beta_{\text{3}}\cdot \text{ORB}\%+\in \end{array}$$ (3)

where ORtg is points scored per 100 possessions, 3PAr is the share of attempts from three-point range, ORB% is offensive rebound percentage, and ε is the error term. Given the time-ordered nature of the data, model interpretation focuses on association. Model diagnostics included variance inflation factor (VIF) analyses to assess multicollinearity and Durbin–Watson statistics to evaluate residual autocorrelation. Given the strong common temporal trends among the predictors, coefficient-level interpretations were made cautiously.

## 3. Results

### 3.1. The era of “safe velocity”

Linear mixed-effects models accounting for repeated observations within NBA franchises confirmed substantial differences between the two eras (Table 1). Compared with the old era, the new era was associated with a 6.02-possession increase in Pace (β = 6.02, SE = 0.20, 95% CI [5.64, 6.41], p < 0.001) and a 0.136 increase in 3PAr (β = 0.136, SE = 0.004, 95% CI [0.127, 0.144], p < 0.001). ORtg was 4.21 points higher (β = 4.21, SE = 0.31, 95% CI [3.60, 4.82], p < 0.001), while TS% increased by 0.024 (β = 0.024, SE = 0.002, 95% CI [0.021, 0.027], p < 0.001). In contrast, TOV% decreased by 0.92 percentage points (β = −0.92, SE = 0.07, 95% CI [−1.06, −0.77], p < 0.001). These findings indicate that the principal differences between the old and new eras remained statistically robust after accounting for within-franchise dependence.

**Table 1:** Comparison of selected performance indicators between the old and new NBA eras using linear mixed-effects models.

Variable	Old Era, 2005–2014 (n = 300), Mean ± SD	New Era, 2015–2025 (n = 330), Mean ± SD	Adjusted difference (New − Old)	SE	95% CI	p-value
**Pace**	91.95 ± 2.62	97.97 ± 2.68	6.02	0.20	5.64 to 6.41	<0.001
**3PAr**	0.223 ± 0.046	0.358 ± 0.064	0.136	0.004	0.127 to 0.144	<0.001
**ORtg**	106.64 ± 3.35	110.85 ± 4.57	4.21	0.31	3.60 to 4.82	<0.001
**TS%**	0.538 ± 0.018	0.562 ± 0.023	0.024	0.002	0.021 to 0.027	<0.001
**TOV%**	13.59 ± 0.92	12.67 ± 0.96	−0.92	0.07	−1.06 to −0.77	<0.001

**Fig 1:**
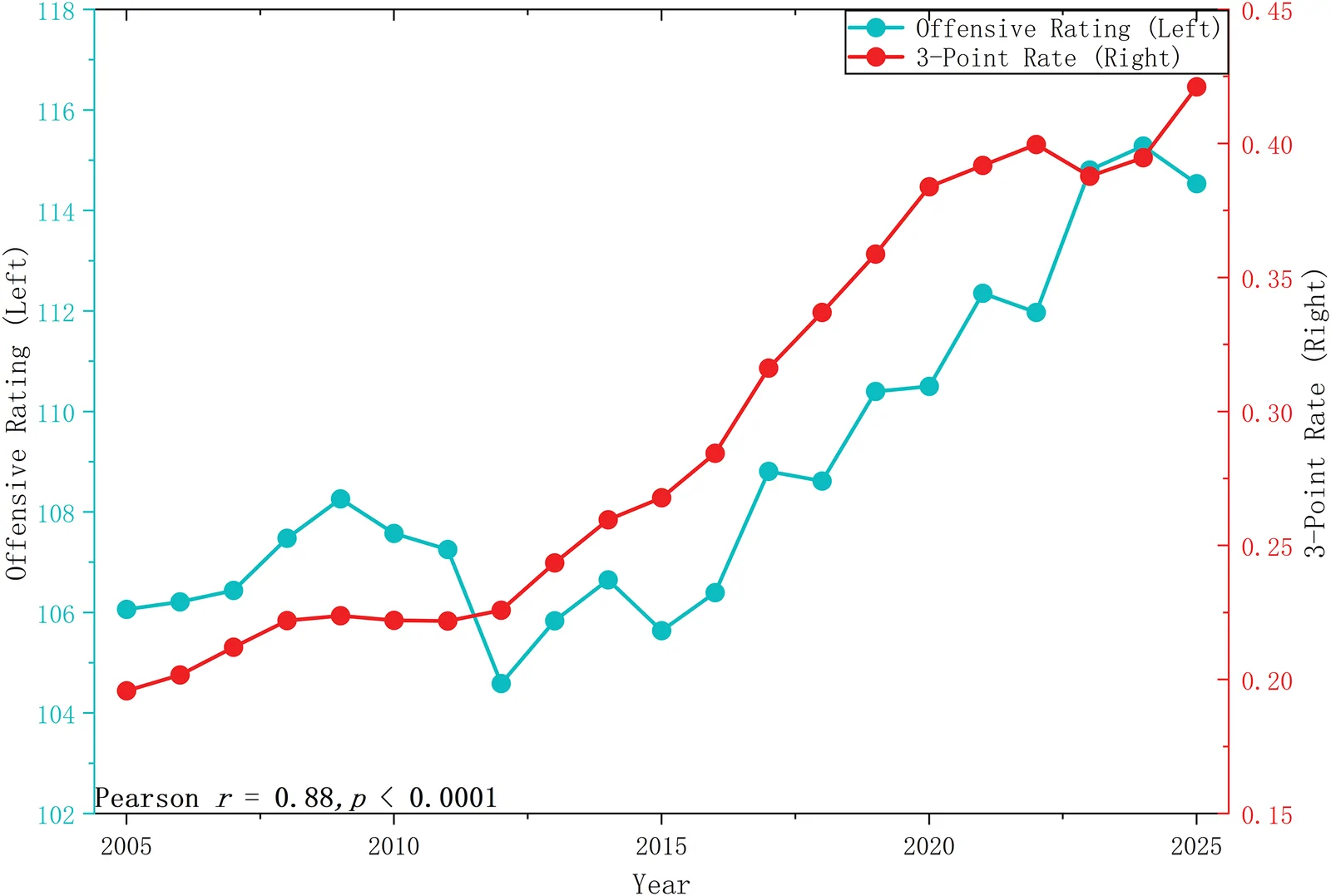
Longitudinal trends and co-movement between Offensive Rating (ORtg) and 3-Point Attempt Rate (3PAr) in the NBA from 2005 to 2025. Note: ORtg denotes Offensive Rating (points per 100 possessions), and 3PAr denotes three-point attempt rate. The Pearson correlation coefficient (r) summarizes the overall association between the two series across seasons. Because both variables exhibit strong upward trends over time, the correlation should be interpreted as descriptive of co-movement rather than as evidence of a causal relationship. Because each point represents the observed league average for an entire season rather than a sampled estimate, confidence intervals are not shown.

To provide broader context, Fig 1 presents the time series of Offensive Rating (ORtg) and 3-Point Attempt Rate (3PAr) from 2005 to 2025. Both variables increased over time, and the two series show clear co-movement. The Pearson correlation between ORtg and 3PAr is strong (r = 0.88, p < 0.001); however, this association may partly reflect shared temporal trends in the modern NBA. Overall, the results indicate that the period characterized by higher pace was also accompanied by higher offensive output (ORtg + 4.21) and greater reliance on three-point attempts (3PAr + 0.136). Across the Table 1 models, marginal R^2^ values ranged from 0.189 to 0.590 and conditional R^2^ values ranged from 0.242 to 0.624, indicating that era explained a substantial proportion of variation in Pace and 3PAr, while franchise-level heterogeneity provided additional explanatory information across outcomes.

### 3.2. Spatial polarization in shot location

**Fig 2:**
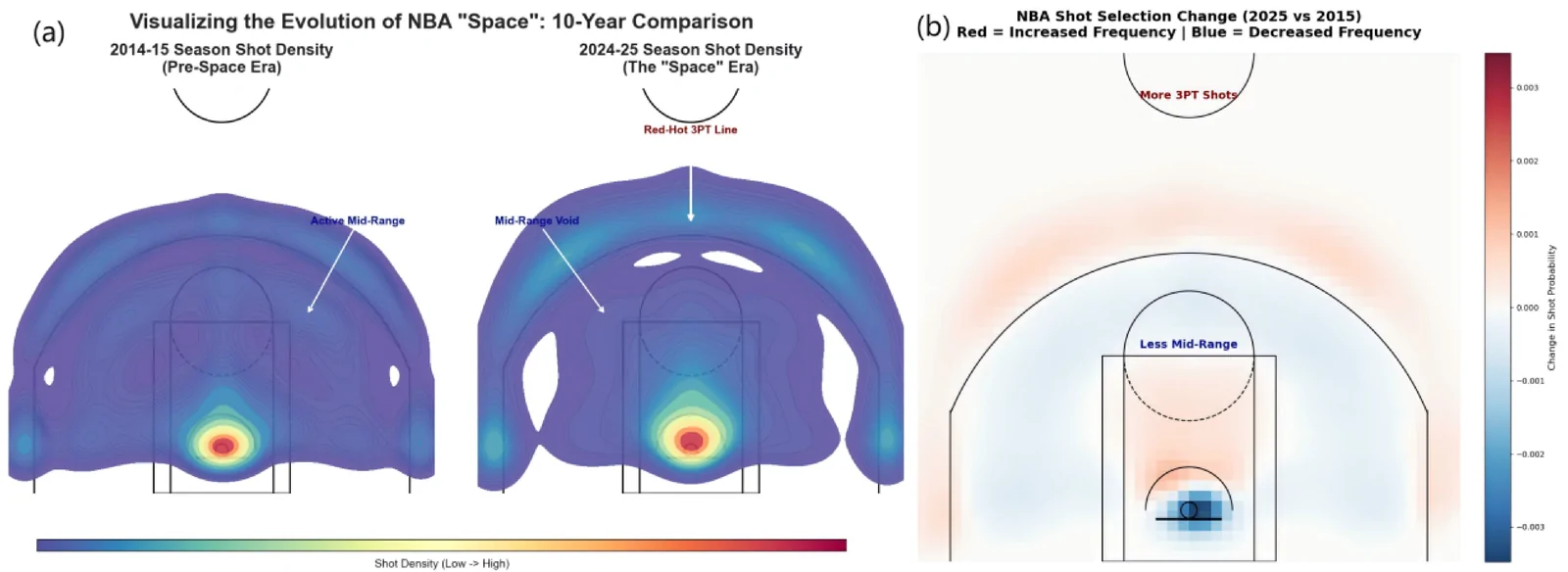
Spatiotemporal evolution of NBA shooting density (2014–2025). (a) Shot-density heatmaps illustrating a shift toward greater concentration at the rim and beyond the three-point arc. (b) Differential heatmap (*Density*_2025_ - *Density*_2015_) highlighting decreased mid-range density (blue) and increased three-point density (red). Note: Data derived from nba_api (N2015 = 205,570; N2025 = 219,527). The redistribution is summarized by an increase in the Spatial Allocation Index (SAI) from 58.98% to 69.99%.

Fig 2 illustrates changes in NBA shot-location density over time, moving from a relatively diffuse distribution to a more concentrated pattern. In Fig 2(a), the 2014–15 season shows substantial shot volume in mid-range areas, alongside attempts at the rim and from three-point range. In contrast, the 2024–25 heatmap shows greater concentration of attempts in two regions: the restricted area and areas beyond the three-point arc. The differential heatmap in Fig 2(b) (Density_2025_ − Density_2015_) visualizes this redistribution, with a clear decline in mid-range density and a corresponding increase in three-point density.

Table 2 provides a simple numerical summary of these shifts. Mid-range attempt share decreased from 41.01% to 30.01% (Δ=−11.00 percentage points), between 2014–15 and 2024–25. Over the same period, the three-point share increased by 15.31 percentage points, reaching 42.12% of all field-goal attempts. Rim attempts showed a modest decline in share (−4.30 percentage points), consistent with a larger reallocation toward perimeter volume.

**Table 2:** Comparison of shot distribution and Spatial Allocation Index (2014–15 vs. 2024–25).

Season	Total Shots (N)	Rim %	Mid-Range %	3-Point %	SAI^a^
**2014–15**	205,570	32.17	41.01	26.81	58.98
**2024–25**	219,527	27.87	30.01	42.12	69.99
**Change (∆** percentage points)	--	−4.30	−11.00	+15.31	+11.01

Consistent with these changes, the Spatial Allocation Index (SAI)—defined as Rim% + 3-Point%—increased from 58.98% to 69.99% (Δ = +11.01 percentage points). Because SAI captures shot-location allocation rather than shot-making efficiency, the increase should be interpreted as a stronger concentration of shot volume in the rim and three-point zones, not as direct evidence of improved conversion rates. The opposing movement of mid-range and three-point shares appears systematic across the period, and is consistent with the broader league shift toward perimeter-oriented shot portfolios described in recent literature [1].

### 3.3. Descriptive evaluation of the spatial allocation index

After harmonizing franchise identifiers, the association analyses included 630 team-season observations from 30 NBA franchises. Descriptive Pearson correlations showed that SAI was strongly associated with eFG% (r = 0.707), moderately associated with TS% (r = 0.639) and ORtg (r = 0.572), and only weakly associated with winning percentage (r = 0.084).

Table 3 summarizes the associations between the Spatial Allocation Index (SAI) and offensive performance indicators. The mixed-effects models produced similar results after accounting for repeated observations within franchises. SAI was positively associated with eFG% (β = 0.00235, SE = 0.00009, 95% CI [0.00219, 0.00252], p < 0.001), TS% (β = 0.00188, SE = 0.00008, 95% CI [0.00172, 0.00205], p < 0.001), and ORtg (β = 0.318, SE = 0.017, 95% CI [0.284, 0.352], p < 0.001). By contrast, the association between SAI and winning percentage was not statistically significant after accounting for within-franchise dependence (β = 0.00123, SE = 0.00069, 95% CI [−0.00013, 0.00259], p = 0.076). Thus, greater shot-location polarization was associated with higher offensive efficiency, but it was not independently associated with winning percentage in the dependence-adjusted analysis.

**Table 3:** Associations between the Spatial Allocation Index and offensive performance indicators.

Outcome	Pearson r	Mixed-model coefficient β	SE	95% CI	Adjusted p-value
**eFG%**	0.707	0.00235	0.00009	0.00219 to 0.00252	<0.001
**TS%**	0.639	0.00188	0.00008	0.00172 to 0.00205	<0.001
**ORtg**	0.572	0.318	0.017	0.284 to 0.352	<0.001
**Winning percentage**	0.084	0.00123	0.00069	−0.00013 to 0.00259	0.076

These associations should be interpreted cautiously because eFG%, TS%, and ORtg are partly sensitive to the same rim-versus-perimeter shot composition captured by SAI. Accordingly, the observed relationships provide evidence of practical association rather than fully independent construct validation. Model-fit indices were consistent with these associations: marginal R^2^ was highest for eFG% (0.511) and TS% (0.417), whereas the winning-percentage model showed very limited variance explained by SAI alone (marginal R^2^ = 0.005).

### 3.4. Association between perimeter volume and offensive output

**Fig 3:**
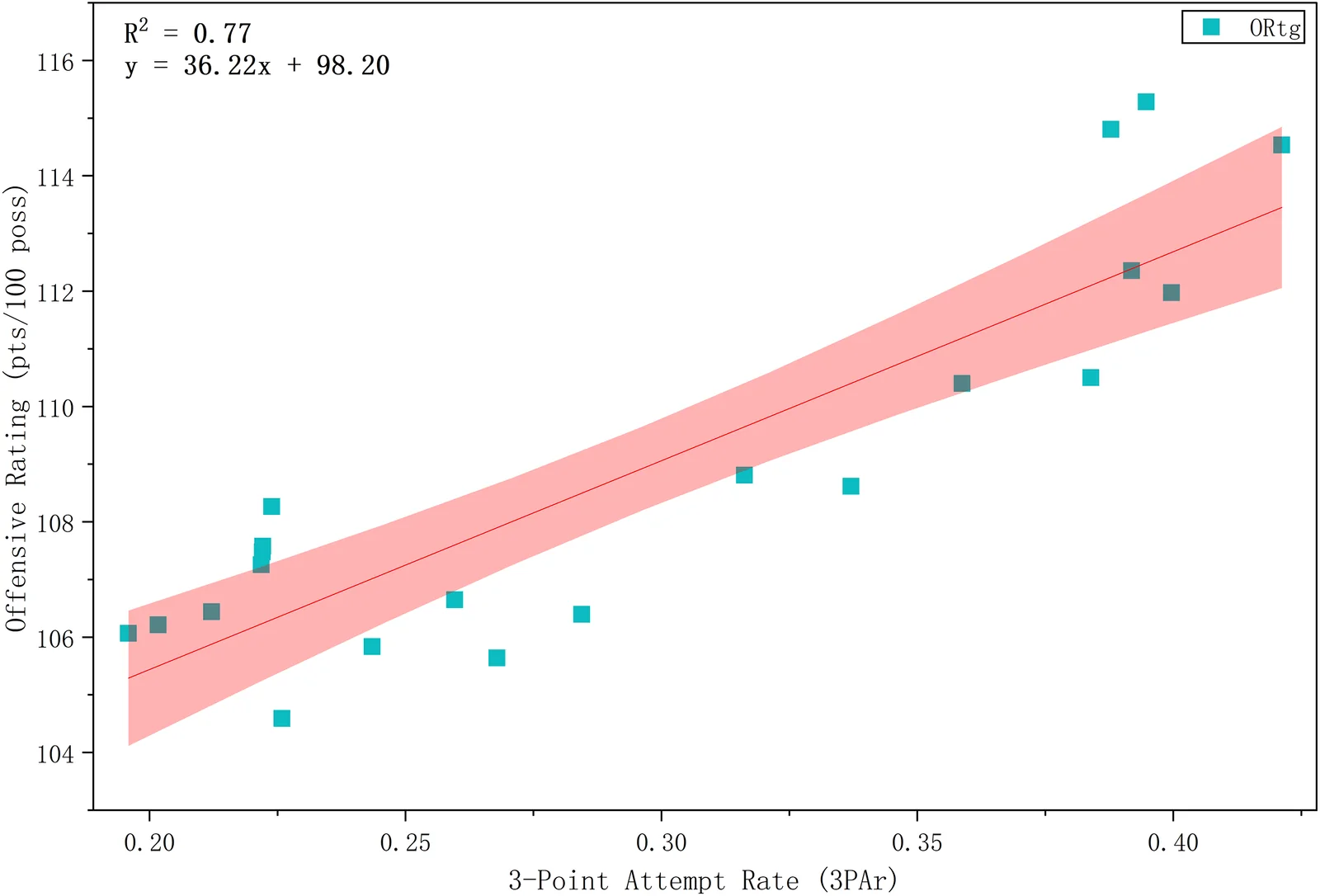
Linear regression analysis of 3-Point Attempt Rate (3PAr) vs. Offensive Rating (ORtg). Note: The solid line shows the fitted regression, and the shaded region indicates the 95% confidence interval. The regression summarizes association across observations and does not, by itself, establish a causal effect.

The league-wide rise in three-point volume coincides with higher offensive output, and Fig 3 summarizes this relationship using a linear regression of Offensive Rating (ORtg) on 3-Point Attempt Rate (3PAr). The fitted model shows a strong positive association, with an R^2^ of 0.77 indicating that a large share of the variation in ORtg across observations is captured by differences in three-point attempt rate.

The estimated regression equation is ORtg = 98.20 + 36.22 × 3PAr. Interpreted descriptively, the slope (36.22) implies that a 0.10 (10 percentage-point) increase in 3PAr is associated with an increase of approximately 3.6 points in ORtg. Given that many NBA games are decided by small margins, differences of this magnitude are likely to be meaningful in practice.

Fig 3 also reports the 95% confidence interval around the fitted line. The interval remains relatively narrow across the observed range of 3PAr, suggesting that the association is not driven by a small number of extreme observations and is broadly consistent across the data. Because both 3PAr and ORtg have increased over time, this relationship should be interpreted as co-movement and association, and not as evidence that changes in three-point rate alone cause improvements in offensive efficiency. Overall, the results are consistent with the view that perimeter-oriented shot allocation is closely aligned with higher possession-based scoring output in the modern NBA.

### 3.5. Pace dynamics around the 2018–19 shot-clock reset

**Fig 4:**
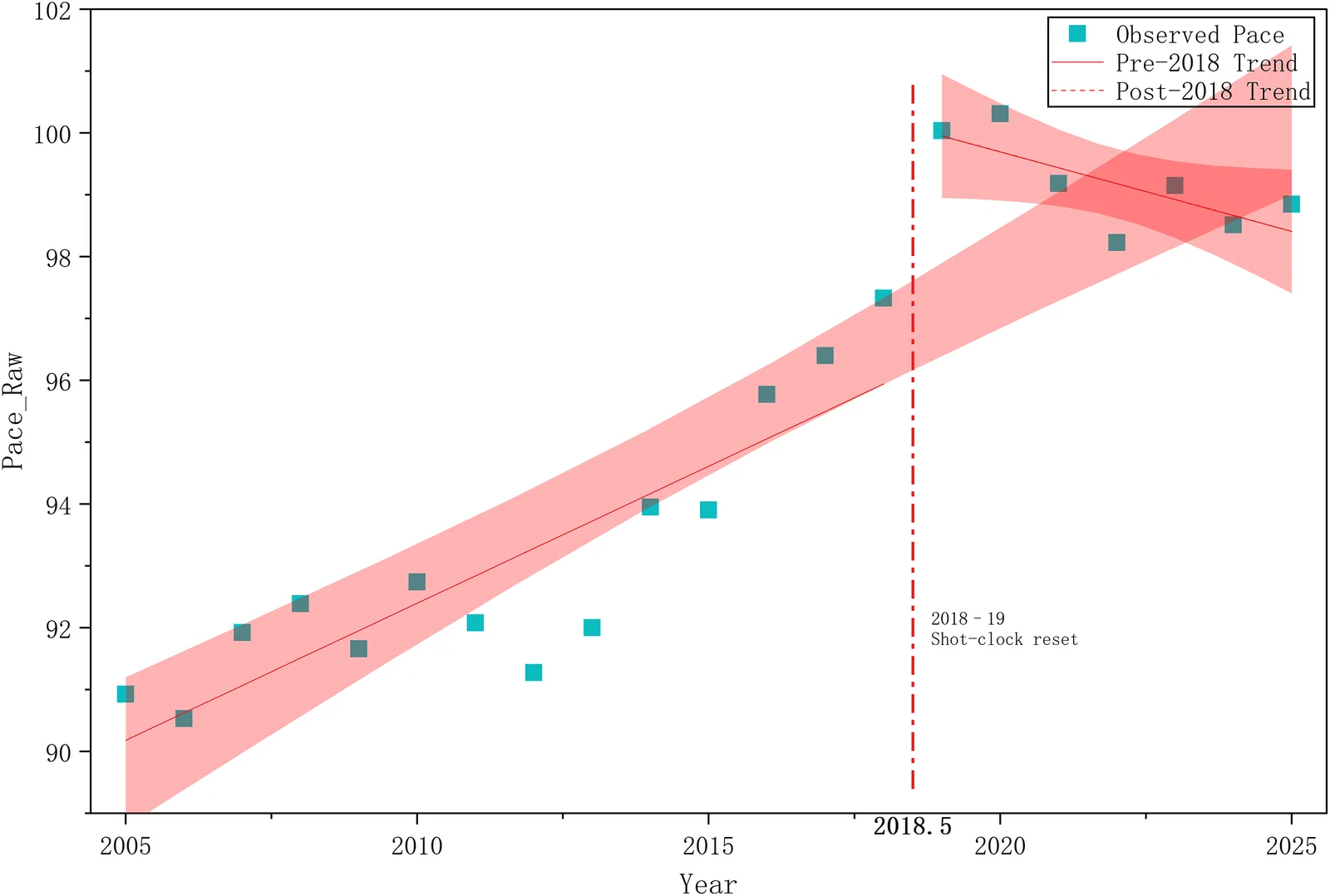
Interrupted time-series analysis of league-average NBA Pace from 2005 to 2025. Note: The vertical dashed line indicates the implementation of the 2018–19 offensive-rebound shot-clock reset. Trends before and after the intervention were estimated using segmented regression. The shaded regions represent the 95% confidence intervals around the fitted pre-intervention and post-intervention trends. Because the analysis is based on a single league-level time series, results should be interpreted as evidence of temporal alignment and trend change rather than definitive causal estimates of the isolated rule effect.

To examine changes in league tempo around the 2018–19 rule adjustment (the 14-second shot-clock reset following an offensive rebound), we estimated a segmented regression model within an interrupted time-series (ITS) framework. This approach allows the Pace series to be described in terms of changes in level and trend associated with a predefined time point, while recognizing the observational nature of the data. As shown in Fig 4, the Pace trajectory is not well characterized by a single linear trend across the full period.

The segmented regression model explained a large proportion of variance in league-average Pace (R^2^ = 0.935; adjusted R^2^ = 0.924). Using HAC/Newey–West standard errors (max lag = 2) and t-based inference with 17 residual degrees of freedom, the pre-2018 trend was positive and statistically significant (β_1_ = 0.443, SE = 0.074, t(17) = 5.95, 95% CI [0.286, 0.600], p < 0.001), corresponding to an average increase of approximately 0.44 possessions per season before the 2018–19 breakpoint. The 2018–19 season was associated with an immediate upward level change in Pace (β_2_ = 4.268, SE = 0.709, t(17) = 6.02, 95% CI [2.772, 5.763], p < 0.001). The post-breakpoint trend change was negative (β_3_ = −0.700, SE = 0.106, t(17) = −6.60, 95% CI [−0.923, −0.476], p < 0.001), yielding a net post-2018 slope of approximately −0.26 possessions per season. The Durbin–Watson statistic was 1.058, suggesting the presence of some residual autocorrelation and supporting the use of HAC/Newey–West standard errors for statistical inference. A sensitivity analysis indicated that, with 17 residual degrees of freedom and a two-sided α level of 0.05, the minimum detectable effect at 80% power was approximately 0.22 possessions per season for the pre-intervention slope and 2.11 possessions for the immediate level change. Both observed effects exceeded these thresholds (β_1_ = 0.443 and β_2_ = 4.268, respectively).

Taken together, these results indicate that the 2018–19 season was temporally aligned with a clear level shift and trend reversal in the league Pace series. Because the design uses a single league-level time series and lacks a control group, these estimates should be interpreted as temporal alignment and structural description rather than as an isolated causal effect of the shot-clock rule change.

As a sensitivity analysis, additional segmented-regression models were estimated using alternative placebo breakpoints in adjacent seasons. These alternative specifications produced weaker level-change estimates and poorer overall model fit than the primary 2018–19 specification, suggesting that the 2018–19 season provides a reasonable and empirically supported breakpoint for describing changes in the league-average Pace series.

### 3.6. Correlates of offensive rating

Although descriptive patterns indicate that league Pace and offensive output increased over time, assessing their relative associations with efficiency requires multivariate analysis. Table 4 reports the results of a multiple linear regression model with Offensive Rating (ORtg) as the dependent variable. The model explained a substantial proportion of variance in ORtg (R^2^ = 0.859; adjusted R^2^ = 0.834; F(3,17) = 34.51, p < 0.001), suggesting that the included predictors capture several core dimensions associated with offensive performance at the league level.

**Table 4:** Multiple linear regression analysis of factors associated with Offensive Rating.

Predictor	*B*	*S.E.*	*95% CI*	β	*t*-value	*p*-value
**Constant**	34.82	43.95	−57.91 to 127.55	—	0.79	0.439
**3PAr**	42.3	14.77	11.13 to 73.47	1.028	2.86	0.011
**ORB%**	1.06	0.37	0.28 to 1.84	0.627	2.88	0.01
**Pace**	0.37	0.43	−0.54 to 1.28	0.388	0.85	0.405

**Note:** Variance inflation factors (VIFs) were 15.52 (3PAr), 5.71 (ORB%), and 24.86 (Pace).

Diagnostic analyses indicated substantial multicollinearity among the league-level predictors. The VIF values were 15.52 for 3PAr, 5.71 for ORB%, and 24.86 for Pace. This collinearity likely reflects the strong shared temporal trends among these variables across the 21-season study period. Accordingly, the individual regression coefficients should be interpreted as conditional descriptive associations rather than as stable estimates of independent predictor effects.

Within this specification, Pace is not statistically significant once other variables are included (B = 0.37, p = 0.405). This result indicates that, conditional on shot-allocation patterns and possession-related outcomes, variation in tempo is not independently associated with ORtg in this model. Importantly, this finding should be interpreted as a conditional association rather than as evidence that tempo is unimportant for offensive success more broadly.

By contrast, three-point attempt rate (3PAr) shows a positive and statistically significant association with ORtg (B = 42.3, p = 0.011). Interpreted descriptively, a higher share of three-point attempts is aligned with higher possession-based scoring output. Offensive rebound percentage (ORB%) is also positively associated with ORtg (B = 1.06, p = 0.01), indicating that extending possessions through offensive rebounding remains relevant even in perimeter-oriented offensive structures.

These regression results are consistent with prior work highlighting shooting-related indicators and rebounding as key correlates of team performance (Cabarkapa et al., 2022) [12]. Within this league-level model, 3PAr and ORB% retained statistically significant conditional associations with ORtg, whereas Pace did not. However, the substantial multicollinearity among the predictors limits direct comparisons of their relative importance, and the coefficients should be interpreted as conditional associations rather than independent effects. In this context, the increase in the Spatial Allocation Index (SAI) should be understood as reflecting a systematic reorientation of shot portfolios that co-occurs with higher offensive output, rather than as a direct causal driver of efficiency.

## 4. Discussion

### 4.1. Shot-location concentration and strategic interpretation

Fig 2(b) documents a clear redistribution in NBA shot locations over time, with attempts becoming increasingly concentrated near the rim and beyond the three-point line, alongside a continued decline in mid-range usage. This pattern indicates that teams have converged toward a relatively stable configuration of shot allocation at the league level. The rise in the Spatial Allocation Index (SAI) reflects this shift by summarizing the growing share of attempts taken from these two regions.

Rather than reflecting a transient stylistic preference, this redistribution is more plausibly interpreted as a strategic response to changes in the competitive environment. Research suggests that once league-wide shooting proficiency surpasses a critical threshold, perimeter-oriented shot portfolios become increasingly attractive [24]. In the modern NBA, improvements in shooting skill, offensive spacing, and lineup flexibility are consistent with such a setting, in which rim attempts and three-point shots tend to offer comparatively favorable expected outcomes.

At the same time, this convergence is best described as an empirical equilibrium rather than a formal Nash equilibrium. Teams do not coordinate shot-selection strategies explicitly; instead, similar outcomes may emerge through independent adaptation under shared competitive pressures, scouting practices, and information diffusion. Within this framework, the decline in mid-range volume can be understood as a rational adjustment to relative shot value, rather than as a consequence of rule-based constraints.

This interpretation is also consistent with the optimal stopping framework proposed in previous basketball research. Earlier offensive systems often devoted more possession time to searching for marginally better opportunities, potentially at the expense of expected value as the shot clock elapsed [17]. In contrast, the contemporary willingness to attempt three-point shots earlier in possessions aligns with the idea that rational shot-acceptance thresholds should adjust downward as time constraints tighten.

### 4.2. Temporal compression and practical constraints on pace

Fig 4 indicates that the 2018–19 shot-clock reset following offensive rebounds coincides with a marked change in league tempo. By reducing the available decision window after second-chance opportunities, the rule may have encouraged earlier initiation of actions and limited prolonged possession resets.

Following this adjustment, however, league Pace growth slowed and stabilized. This pattern does not necessarily imply the existence of a single, fixed physiological ceiling. Instead, it is more consistent with teams optimizing under a combination of physical, perceptual–cognitive, and strategic constraints. As tempo increases, the marginal benefits of further acceleration may diminish, while associated costs—such as fatigue accumulation, execution errors, or transition vulnerability—may rise more rapidly.

The temporal shift also coincides with changes in player profiles and role demands. Recent literature describes the emergence of a “new basketball body,” characterized by lower body mass and a greater emphasis on agility and movement efficiency rather than static strength [25]. Recent normative data from competitive basketball populations further support this trend by showing that contemporary players increasingly exhibit physical-performance profiles emphasizing speed, agility, and movement efficiency across developmental stages [26]. These attributes may be particularly well suited to repeated high-frequency transitions and the reduced time available after offensive rebounds.

From this perspective, the post-intervention stabilization of Pace (net slope ≈ −0.26) is consistent with a diminishing-returns regime rather than an abrupt performance limit. As research notes, repeated high-intensity actions impose substantial metabolic and musculoskeletal stress [15]. Basketball-specific repeated-sprint training studies likewise demonstrate that sustaining repeated multidirectional high-intensity efforts requires substantial physiological adaptation, supporting the view that progressively faster game tempos may eventually encounter practical physical constraints [27,28]. Teams may therefore balance tempo gains against injury risk and cumulative load [29], a trade-off that aligns with the league’s increasing emphasis on load management. In this sense, the observed pattern reflects strategic moderation rather than absolute constraint.

Finally, the post-2019 period also featured broader tactical consolidation, including near-saturation of three-point usage and increased positional interchangeability. Once major spatial advantages had largely been realized, the incentive to pursue additional increases in pace may have been reduced.

### 4.3. Defensive Spacing and the “efficiency paradox”

A notable pattern in the results is that league Pace increased while turnover percentage declined, producing what we describe as an “efficiency paradox” (Table 1). Under a conventional speed–accuracy framework, higher tempo would be expected to increase error rates. The modern NBA trend departs from this expectation.

One plausible interpretation involves changes in defensive geometry under contemporary spacing. Offensive gains may arise not only from individual skill improvements but also from reducing defensive compactness and constraining the defense’s ability to protect multiple high-value areas simultaneously. Previous research has conceptualized defensive effectiveness in spatial terms, emphasizing a defender’s capacity to reduce offensive value within a localized radius of “spatial influence.” [13] The shot distributions shown in Fig 2 suggest that modern offenses increasingly separate primary threats at the rim and beyond the three-point line, requiring defenses to cover larger effective areas.

Within this context, declining turnover rates may reflect simplified decision environments rather than only improved ball-handling ability. When defenses are stretched across wider distances, passing lanes may become more stable and help-and-recover rotations more predictable. Common actions such as drive-and-kick can therefore be executed with clearer reads and reduced congestion. In this sense, higher tempo may also be sustainable because changes in offensive spacing alter the spatial structure of defensive interactions, potentially reducing the frequency of high-pressure situations and allowing speed and ball security to coexist more readily [13].

### 4.4. Strategic trade-offs in high-tempo offense

Despite its advantages, the modern high-tempo, perimeter-oriented approach entails clear trade-offs. In the present analysis, 3-Point Attempt Rate (3PAr) showed a strong negative association with Offensive Rebound Percentage (ORB%) (r = −0.94), indicating that increased perimeter volume is accompanied by reduced commitment to offensive rebounding. A likely explanation is tactical: teams seeking to maintain tempo and limit transition exposure often prioritize floor balance and rapid defensive retreat over sending multiple players to the offensive glass.

Importantly, increased offensive pace does not imply diminished importance of defense. Previous studies have reported that Defensive Rating remains a significant predictor of winning even in higher-scoring environments [30]. This suggests a layered competitive balance in which pace and spacing may elevate baseline efficiency during the regular season, while defensive organization and possession control continue to constrain outcomes against strong opposition.

The value of pace and perimeter emphasis may also be context dependent. Research examining decisive NBA playoff games (Game 7s) finds that while spacing remains relevant, defensive rebounding and assists are particularly strong predictors of success, and turnovers exert a larger negative influence [31]. Together, these findings imply that modern optimization involves trade-offs: strategies that raise expected value through tempo and three-point volume may come at the cost of rebounding and transition control, and the relative importance of these margins may increase in high-stakes settings.

### 4.5. Strengths and practical applications

This study has several strengths. First, it incorporates data from 21 consecutive NBA regular seasons, providing a long-term perspective on offensive evolution that extends beyond short-term fluctuations and season-specific effects. Second, the analysis integrates both macro-level team performance indicators and micro-level shot-location data, allowing offensive changes to be examined from complementary spatial and temporal perspectives. Third, by combining spatial analysis, interrupted time-series modeling, and multivariate regression, the study captures not only how offensive behavior evolved over time but also how these changes aligned with broader league-level performance trends. Finally, the introduction and descriptive evaluation of the Spatial Allocation Index (SAI) provide a parsimonious indicator for summarizing long-term changes in shot-allocation preferences across seasons.

From a practical perspective, the findings may be relevant for coaches, performance analysts, and player-development practitioners. The observed increase in shot concentration at the rim and beyond the three-point line suggests that contemporary offensive systems increasingly emphasize spatial efficiency and shot-value optimization. In addition, the stabilization of pace following the 2018–19 shot-clock reset indicates that future offensive gains may depend less on further increases in game tempo and more on improving decision-making quality, spacing effectiveness, and shot selection within existing temporal constraints. These findings may assist practitioners in evaluating offensive strategies, identifying player-development priorities, and understanding long-term tactical trends in elite basketball.

## 5. Conclusion

The spatiotemporal evolution of NBA offenses from 2005 to 2025 reflects a substantial shift in how teams distribute shot attempts and manage possessions. Using two decades of team-level indicators and shot-location data, this study documents a league-wide movement toward faster tempo and greater reliance on perimeter attempts, consistent with gradual strategic adaptation under shared competitive constraints.

Across periods, the results do not align with a simple speed–accuracy trade-off at the league level. Pace increased significantly while turnover percentage declined (p < 0.001), a pattern we describe as “safe velocity,” meaning that higher tempo co-occurred with improved possession security. Spatially, mid-range attempts declined sharply, while the Spatial Allocation Index (SAI) rose to 69.99%, indicating that a larger share of attempts was concentrated at the rim and beyond the three-point line. Because SAI captures shot-location allocation rather than shot-making efficiency, this finding should be interpreted as a redistribution of shot volume toward these areas rather than direct evidence of improved conversion.

The interrupted time-series analysis also shows a clear change in the Pace series around the 2018–19 shot-clock reset. The breakpoint is associated with an immediate increase in Pace (β_2_) and a negative change in the post-intervention trend (β_3_), producing a stabilization pattern rather than continued acceleration. Given the single league-level time series, these results are best interpreted as temporal alignment and trend change, suggesting that the rule adjustment may have interacted with ongoing tactical developments and contributed to a period of consolidation in tempo growth.

Taken together, the findings indicate that the modern NBA has moved toward a relatively stable offensive configuration characterized by strong shot-location concentration and a tempo that has recently flattened. As gains from further increases in pace or three-point volume appear less pronounced, future competitive advantages may depend more on higher-order execution factors—such as decision efficiency and shot-creation quality—including the ability to generate advantages under pressure and convert them into high-value attempts.

### 5.1. Limitations and future directions

Several limitations should be considered when interpreting the present findings. First, although the Spatial Allocation Index (SAI) provides a concise summary of shot-allocation tendencies, it does not account for shot difficulty, defensive pressure, player skill level, or expected possession value. Consequently, SAI should be interpreted as a descriptive indicator of spatial preference rather than a direct measure of offensive effectiveness.

Second, the interrupted time-series analysis was conducted using a single league-level time series and therefore cannot isolate the independent causal effect of the 2018–19 shot-clock reset from other concurrent developments in the NBA. Changes in coaching philosophies, player skill profiles, roster construction, and league-wide tactical evolution may also have contributed to the observed trends.

Third, the present study focused on league-level and team-level aggregated statistics. As a result, potential heterogeneity across teams, playing styles, competitive levels, and roster compositions was not explicitly modeled. Because repeated observations originated from the same NBA franchises across seasons, we addressed within-franchise dependence using linear mixed-effects models with franchise-specific random intercepts. Nevertheless, these models primarily accounted for stable between-franchise heterogeneity and did not explicitly model more complex temporal dependence, franchise-specific slopes, roster continuity, coaching changes, or organizational evolution across seasons. Future research may adopt more flexible longitudinal modeling approaches to further examine these sources of variation.

Fourth, the study relied on publicly available NBA Stats shot-location data. Although the spatial classification procedure was fully deterministic and therefore reproducible when identical coordinate thresholds were applied, the accuracy of the underlying shot coordinates could not be independently verified because no external coordinate dataset or validated tracking source was publicly available for cross-validation. Accordingly, the Spatial Allocation Index (SAI) should be interpreted as a reproducible rule-based indicator derived from the published NBA coordinates rather than as a direct validation of the underlying tracking measurements. Future studies may compare NBA Stats coordinates with independent tracking systems or video-based annotations to further evaluate measurement accuracy.

Future studies may integrate player-tracking data, expected-shot-value models, and advanced spatial analytics to further evaluate how offensive spacing, shot quality, and decision-making interact to influence team performance. Longitudinal analyses that incorporate tactical, roster, and contextual variables may also help clarify the mechanisms underlying the long-term evolution of modern basketball offense.

## Supporting information

S1 Data Team-season performance data used in the analyses. (CSV)

S2 Data Team-season shot-allocation and Spatial Allocation Index (SAI) data used in the analyses. (CSV)

S3 File Supplementary materials, including additional methodological details and supplementary figures. (PDF)
