# Supplementary material for: Shot-location polarization and post-2018 pace stabilization in the NBA: Evidence from 21 seasons of team and shot-coordinate data (2004–2025)
Source: PLoS One. 2026 Sep 18;21(9):e0358360. doi: 10.1371/journal.pone.0358360 (PMC13588525; doi:10.1371/journal.pone.0358360)
Supplement: Supplementary file 1 [file pone.0358360.s003.pdf]

Short Supplementary Methods

Supplementary Methods. All analyses use league-season aggregates across 21 NBA seasons (season-ending years 2005–2025; N=21). The season index *t* denotes the ordered season sequence (1–21). ORtg is measured as points scored per 100 possessions. 3PAr denotes three-point attempt rate (3PA/FGA), and ORB% denotes offensive rebounding percentage; Pace is the league pace metric reported at the season level.

S2.1 Year-adjusted (detrended) association between 3PAr and ORtg

Because both three-point attempt rate (3PAr) and offensive rating (ORtg) exhibit strong long-run time trends at the league level, we evaluated whether their association persists beyond shared temporal movement by removing a linear time trend from each series. Specifically, we regressed each variable on the season index (*t*) and retained the unstandardized residuals; we then examined the Pearson correlation between these detrended residuals. The year-adjusted association remained positive and statistically significant ($r=0.562$, $p=0.008$; N=21). This analysis is descriptive and does not establish causality.

S2.2 OLS sensitivity analysis with an explicit time control

To reduce sensitivity to secular trends, we re-estimated the baseline OLS model by adding an explicit time control (season index, *t*). Table S1 reports coefficients from the baseline specification (ORtg~ 3PAr+ORB%+Pace) and the time-controlled specification (ORtg~ 3PAr+ORB%+Pace+*t*). The associations of ORtg with 3PAr and ORB% remained positive in the time-controlled model, whereas Pace was not statistically significant in either specification; the coefficient on t was not statistically significant. Given league-level aggregation and potential remaining time dependence, these results should be interpreted as conditional associations rather than definitive effect estimates.

Table S1 caption + note

Table S1. OLS sensitivity analysis with explicit time control (season index *t*; league-season, N=21). Values are unstandardized coefficients B with standard errors in parentheses; two-sided p-values are reported. Model fit: Baseline R^2=0.859, adjusted R^2=0.834. +*t* R^2=0.863, adjusted R^2=0.829.

Predictor	Baseline *B* (SE)	p	+*t* *B* (SE)	p
ThreePAr	42.304 (14.769)	0.011	56.165 (24.772)	0.038
ORB%	1.064 (0.369)	0.010	0.996 (0.387)	0.020
Pace	0.367 (0.430)	0.405	0.268 (0.458)	0.566
t	—	—	−0.146 (0.207)	0.492
Constant	34.822 (43.948)	0.439	43.451 (46.275)	0.362

Table note: These models use league-season aggregates; coefficients are interpreted as conditional associations and do not identify causal effects. ORB% corresponds to the SPSS variable ORB_pct.

Figure S1 caption

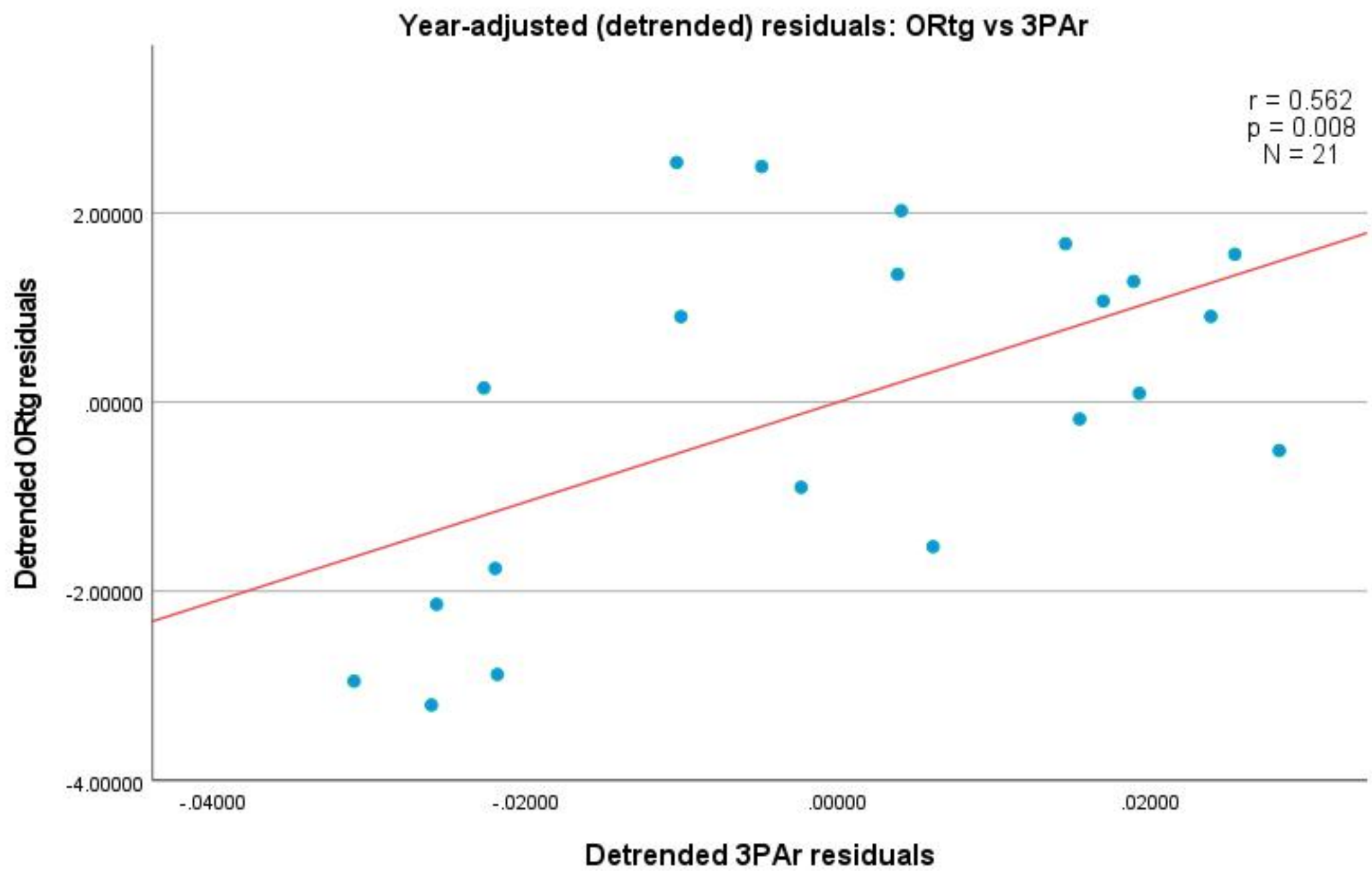

Figure S1. Year-adjusted co-movement between 3PAr and ORtg (detrended residuals).

Note: Residuals were obtained by separately regressing league-season ORtg and 3PAr on the season index (t) and retaining unstandardized residuals. The scatterplot shows the association between these detrended residuals (Pearson r=0.562, p=0.008; N=21). This visualization is descriptive and does not establish causality. Here, *t* is the ordered season index (1–21).

Figure S2 caption (recommend one extra sentence)

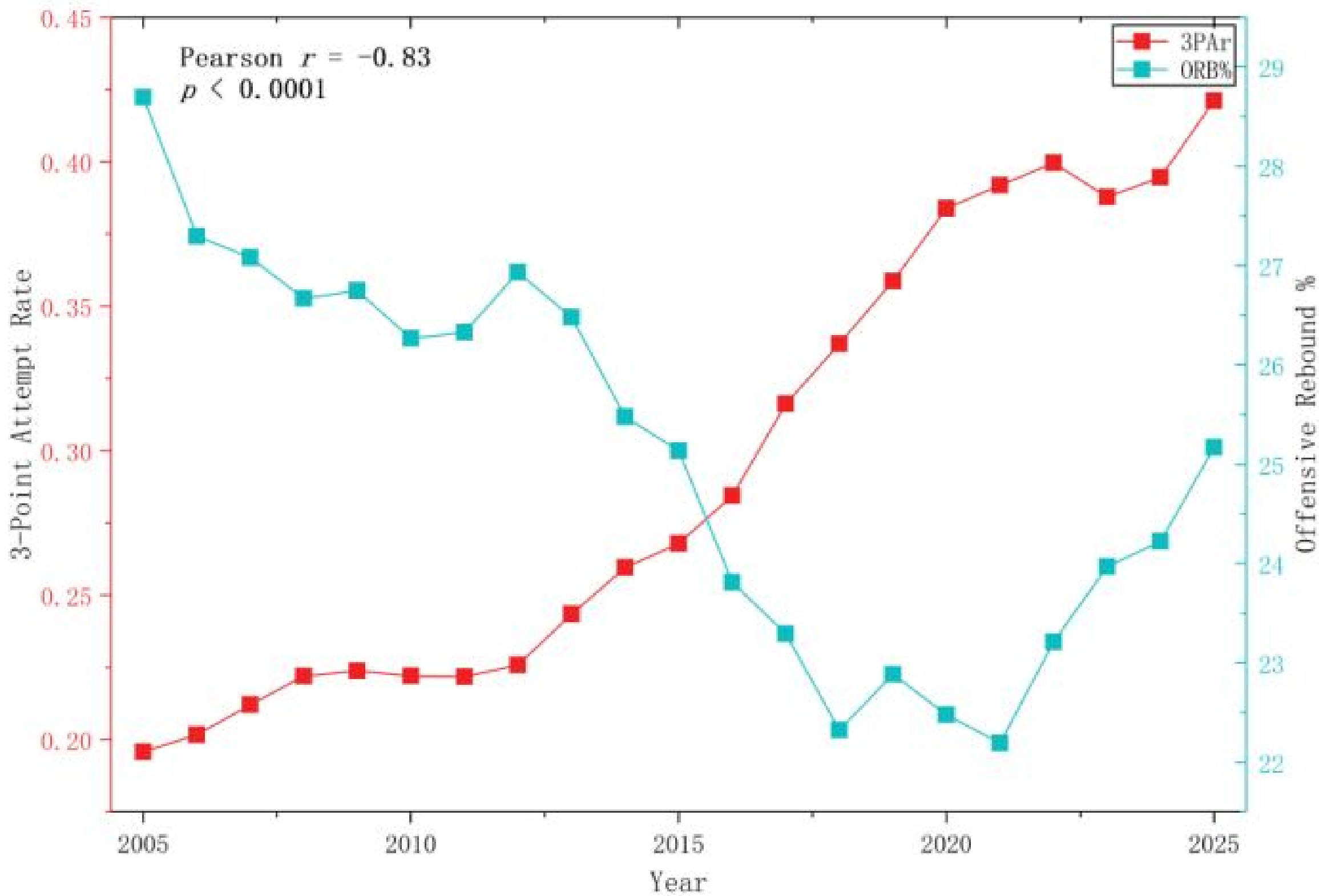

Figure S2. Co-movement between three-point attempt rate (3PAr) and offensive rebounding rate (ORB%), 2005–2025.

Note: The series show an inverse association at the league-season level (Pearson $r=-0.83$, $p<0.0001$; $N=21$). Axes are scaled separately; the figure is intended for descriptive comparison of trends and does not establish causal mechanisms.
