# Supplementary material for: Shot-location polarization and post-2018 pace stabilization in the NBA: Evidence from 21 seasons of team and shot-coordinate data (2004–2025)
Source: PLoS One. 2026 Sep 18;21(9):e0358360. doi: 10.1371/journal.pone.0358360 (PMC13588525; doi:10.1371/journal.pone.0358360)
Supplement: Supplementary file 2 [file pone.0358360.s005.docx]

Response to Reviewers

Manuscript ID: PONE-D-26-10048

Title: Shot-location polarization and post-2018 pace stabilization in the NBA: evidence from 21 seasons of team and shot-coordinate data (2004–2025)

Dear Academic Editor and Reviewers,

We sincerely thank the Academic Editor and the reviewers for their careful evaluation of our manuscript and for their constructive and insightful comments. We have revised the manuscript extensively in response to all suggestions. The revisions have improved methodological transparency, statistical reporting, theoretical justification, and overall clarity.

All revisions are marked using tracked changes in the revised manuscript. Below, we provide a detailed point-by-point response to each comment, including the locations of the main changes.

============================================================

EDITOR COMMENTS

============================================================

Comment:

Please provide non-author contact information for data access and clarify long-term data availability.

Response:

Thank you for this important recommendation. We revised the Data Availability Statement to clarify data sources, data-processing procedures, and long-term availability of the derived dataset. In line with the PLOS Data Policy, we designated a non-author institutional contact to handle future data requests:

• Institution/Office: Capital Normal University (首都师范大学)

• Email: 2243702359@cnu.edu.cn

• Phone/URL: Not applicable (email contact provided)

In addition, the derived season-level dataset used for analyses, together with variable definitions and data-processing procedures, is provided in the Supporting Information to ensure persistent access.

Changes in manuscript:

Data Availability Statement revised (Page 22). Table 3 (Page 12), Table 4 (Page 15), and Figure 4 (Page 15) provide the key derived results; the derived dataset is provided in the Supporting Information.

============================================================

REVIEWER #1

============================================================

Comment 1:

The conceptual justification and validation of the Spatial Allocation Index (SAI) require improvement.

Response:

We agree. We expanded the conceptual justification to clarify that SAI is a descriptive measure of shot-allocation structure rather than a direct measure of offensive effectiveness. To evaluate its practical relevance, we added validation analyses examining correlations between SAI and Offensive Rating (ORtg), True Shooting Percentage (TS%), effective Field Goal Percentage (eFG%), and Winning Percentage (Win%). The results support the construct validity of SAI as an indicator of modern shot-allocation patterns.

Changes in manuscript:

Section 2.3.1 expanded; new validation subsection and Table 3 added (Section 3.3; Page 12).

Comment 2:

Provide additional details regarding NBA API extraction and preprocessing procedures.

Response:

We added methodological detail describing data acquisition, inclusion criteria, preprocessing workflow, and spatial classification procedures to improve reproducibility and transparency.

Changes in manuscript:

Section 2.1 Data Acquisition and Preprocessing revised (Methods section).

Comment 3:

The interrupted time-series model lacks confidence intervals, autocorrelation diagnostics, and justification for the selected breakpoint.

Response:

We substantially expanded the interrupted time-series (ITS) analysis and reporting. We now report standard errors, 95% confidence intervals, exact p-values, model fit indices, and Durbin–Watson statistics. HAC/Newey–West standard errors are used for inference. We also included a sensitivity analysis using alternative placebo breakpoints in adjacent seasons to evaluate robustness.

Changes in manuscript:

Sections 2.3.2 and 3.5 revised; Figure 4 updated (Page 15).

Comment 4:

Causal interpretations should be softened.

Response:

We revised the manuscript throughout to emphasize temporal alignment and association rather than causal inference, and explicitly noted the observational nature of the design and the absence of a control group.

Changes in manuscript:

Revisions throughout Abstract/Results/Discussion; Figure 4 note updated (Page 15); Limitations expanded (Discussion).

Comment 5:

The concept of “safe velocity” requires stronger justification.

Response:

We clarified that “safe velocity” is used as a descriptive interpretation of observed league trends rather than a formally validated theoretical construct. Alternative explanations and study limitations are discussed.

Changes in manuscript:

Section 3.1 and Discussion revised.

Comment 6:

Heatmaps require stronger quantitative interpretation.

Response:

We clarified that the heatmaps serve primarily as descriptive visualizations of shot-distribution changes. Their interpretation is now supported by quantitative analyses presented in Table 2 and the SAI results.

Changes in manuscript:

Section 3.2 revised; Table 2 and SAI results support the quantitative interpretation.

Comment 7:

References use mixed citation styles.

Response:

All references were reformatted according to PLOS ONE (Vancouver-style) reference requirements.

Changes in manuscript:

Reference list fully revised.

Comment 8:

Add a discussion of strengths and practical applications.

Response:

A dedicated subsection discussing study strengths and practical implications for coaches, analysts, and player-development practitioners has been added.

Changes in manuscript:

New Section 4.5 (Strengths and Practical Applications) added (Discussion).

============================================================

REVIEWER #2

============================================================

Comment 1:

Provide stronger justification for the analytical design and league-level sample.

Response:

We clarified the rationale for using complete-population NBA league data and discussed the strengths and limitations of league-level analyses.

Changes in manuscript:

Methods and Limitations revised.

------------------------------------------------------------

Comment 2:

Provide confidence intervals, exact p-values, and additional statistical diagnostics.

Response:

We added confidence intervals and diagnostics where appropriate, including adjusted R² values and Durbin–Watson statistics, and clarified interpretive limits due to the league-level time-series structure.

Changes in manuscript:

Table 3 (Page 12), Table 4 (Page 15), and Section 3.5 (Page 15) revised.

Comment 3:

The interrupted time-series model should report HAC/Newey–West standard errors and sensitivity analyses.

Response:

HAC/Newey–West standard errors are now reported. We also included placebo-breakpoint sensitivity analyses in adjacent seasons to evaluate the robustness of breakpoint selection.

Changes in manuscript:

Sections 2.3.2 and 3.5 revised; Figure 4 updated (Page 15).

Comment 4:

Figures should include confidence intervals or uncertainty estimates.

Response:

Figures were revised to include confidence information where appropriate. Figure 4 includes a 95% confidence band around the pre-intervention trend and an explanatory note regarding interpretation.

Changes in manuscript:

Figure 4 updated (Page 15).

Comment 5:

Table 3 and Table 4 require more complete statistical reporting.

Response:

Table 3 was expanded and supplemented with explanatory notes. Table 4 reports model-level statistics including R², adjusted R², F-statistics, and Durbin–Watson diagnostics.

Changes in manuscript:

Table 3 (Page 12) and Table 4 (Page 15) revised.

Comment 6:

Several recommended references should be considered.

Response:

The suggested references were reviewed and incorporated where relevant in the Introduction/Methods/Discussion.

Changes in manuscript:

References and in-text citations updated accordingly.

Comment 7:

English language and typographical issues require correction.

Response:

The manuscript was proofread and edited to improve clarity, consistency, and readability. Identified typographical and formatting errors were corrected.

Changes in manuscript:

Revisions made throughout the manuscript.

============================================================

ADMINISTRATIVE UPDATES

============================================================

In addition to the scientific revisions described above, the affiliation information for the first author (Shuming Cui) has been updated to reflect the current institutional affiliation. We also corrected the corresponding author information in the submission system to ensure consistency with the manuscript: the corresponding author is Zhenxing Yang. These administrative updates do not affect the study design, data collection procedures, analyses, results, interpretation, or conclusions.

============================================================

We sincerely thank the Academic Editor and Reviewers for their valuable comments and suggestions. We believe that the revised manuscript has been substantially strengthened and we respectfully submit it for further consideration.

Sincerely,

Shuming Cui and co-authors
