# Supplementary material for: Shot-location polarization and post-2018 pace stabilization in the NBA: Evidence from 21 seasons of team and shot-coordinate data (2004–2025)
Source: PLoS One. 2026 Sep 18;21(9):e0358360. doi: 10.1371/journal.pone.0358360 (PMC13588525; doi:10.1371/journal.pone.0358360)
Supplement: Supplementary file 3 [file pone.0358360.s006.docx]

# Response to Reviewers

**Manuscript ID:** PONE-D-26-10048R1

**Title:** Shot-location polarization and post-2018 pace stabilization in the NBA: evidence from 21 seasons of team and shot-coordinate data (2004–2025)

## Dear Academic Editor and Reviewers,

We sincerely thank the Academic Editor and both reviewers for the careful evaluation of our manuscript and for the constructive suggestions provided during the previous review round. The comments were extremely helpful in improving the statistical rigor, methodological transparency, and overall presentation of the manuscript.

In this revised version, we carefully addressed every comment. The principal revisions include:

- replacing analyses affected by repeated observations with linear mixed-effects models;
- revising the interrupted time-series (ITS) analysis using finite-sample t-based inference while retaining HAC/Newey–West standard errors;
- correcting the inconsistency between Equation 3 and Table 4;
- reporting variance inflation factors (VIFs);
- expanding the description of the shot-coordinate data source and classification procedure;
- clarifying the interpretation and limitations of the Spatial Allocation Index (SAI);
- correcting all broken cross-references and reference-style inconsistencies; and
- revising the Data Availability Statement to ensure consistency with the manuscript and PLOS ONE requirements.

All modifications have been incorporated into the revised manuscript and are highlighted in the tracked-changes version.

Below we provide a detailed point-by-point response.

# Response to Reviewer #1

We sincerely thank Reviewer #1 for the positive evaluation of our revised manuscript and for the careful reading.

## Comment 1

In some cases you are mixing reference styles (APA and between square brackets).

### Response

Thank you for identifying this issue. We carefully reviewed the entire manuscript and standardized all citations to the journal's numbered reference format. All remaining APA-style citations have been removed to ensure consistency throughout the manuscript.

## Comment 2

"Error! Reference source not found."

### Response

Thank you for noting this formatting problem.

The manuscript was carefully checked and all broken Word cross-reference fields were corrected. We refreshed the document fields, verified all in-text citations against the reference list, and confirmed that no unresolved cross-reference errors remain in the revised manuscript.

We appreciate the reviewer for identifying this issue.

# Response to Reviewer #2

We sincerely thank Reviewer #2 for the detailed and thoughtful evaluation. We greatly appreciate the recognition that the manuscript has substantially improved. The remaining comments have been carefully addressed as described below.

## Comment 1

Table 1 and Table 3 treat repeated observations from NBA franchises as statistically independent.

### Response

We fully agree.

To account for repeated observations from the same NBA franchises across seasons, all analyses previously based on independent-sample tests were replaced with linear mixed-effects models using franchise as a random intercept.

Specifically,

- Table 1 has been completely reanalyzed using linear mixed-effects models;
- Table 3 has likewise been reanalyzed using mixed-effects models;
- the corresponding Methods section has been expanded to describe the statistical model;
- all Results have been updated accordingly; and
- the Limitations section now explicitly explains how within-franchise dependence was addressed and acknowledges the remaining limitations of this modeling strategy.

Importantly, after accounting for repeated observations, the previously marginal association between SAI and winning percentage was no longer statistically significant (β = 0.00123, p = 0.076), whereas the remaining principal findings remained unchanged.

These revisions substantially improve the statistical validity of the manuscript.

## Comment 2

Equation 3 specifies four predictors whereas Table 4 reports three predictors.

### Response

Thank you for identifying this inconsistency.

The inconsistency resulted from an earlier model-development stage in which turnover percentage (TOV%) had been evaluated but was subsequently removed from the final regression model because of substantial multicollinearity and limited additional explanatory value.

Equation 3 has been revised to match the final model reported in Table 4. The regression model now consistently includes:

- Pace,
- 3-Point Attempt Rate (3PAr), and
- Offensive Rebound Percentage (ORB%).

The Methods, equation, and regression results are now fully consistent.

## Comment 3

Report the variance inflation factors (VIFs).

### Response

Thank you for this suggestion.

Variance inflation factors have now been explicitly reported in both the Results section and the note accompanying Table 4.

The VIF values are:

- 3PAr = 15.52
- ORB% = 5.71
- Pace = 24.86

The Results section further explains that these large values reflect strong shared temporal trends among league-level predictors and that regression coefficients should therefore be interpreted as conditional descriptive associations rather than independent causal effects.

## Comment 4

Provide additional information regarding the shot-coordinate data source and reliability.

### Response

Thank you for this valuable suggestion.

The Data Acquisition section has been expanded substantially.

Specifically, we now clarify that:

- shot coordinates were retrieved from the publicly accessible NBA Stats API via the ShotChartDetail endpoint;
- the NBA does not publicly document the underlying coordinate acquisition procedures or measurement uncertainty;
- identical spatial classification thresholds were applied consistently across all seasons; and
- because the classification procedure is deterministic, identical coordinates always produce identical spatial categories, although external validation against an independent coordinate source is not currently available.

These additions improve methodological transparency while accurately reflecting the publicly available information.

## Comment 5

The validation of SAI partly reflects shared mathematical structure with eFG%, TS%, and ORtg.

### Response

We agree with this observation.

The revised manuscript now explicitly acknowledges that the observed associations should be interpreted cautiously because eFG%, TS%, and ORtg are partly influenced by the same rim-versus-perimeter shot composition represented by SAI.

Accordingly, we now describe these analyses as evidence of practical association rather than independent construct validation.

## Comment 6

Statistical inference for the interrupted time-series analysis appears to rely on large-sample normal inference despite only 21 observations.

### Response

Thank you for this important statistical observation.

We agree that finite-sample inference is more appropriate for the interrupted time-series model.

Accordingly, while retaining HAC/Newey–West standard errors to account for serial correlation and heteroskedasticity, we revised the statistical inference to use a t distribution with 17 residual degrees of freedom.

Specifically,

- the Methods section now explicitly describes the finite-sample inference procedure;
- the Results section has been updated with revised confidence intervals and p-values;
- the interpretation of the interrupted time-series analysis remains unchanged.

The substantive conclusions are unaffected, but the statistical reporting is now more appropriate for the available sample size.

## Comment 7

Post-intervention uncertainty should be shown in Figure 4.

### Response

Thank you for this suggestion.

Figure 4 has been revised to display 95% confidence intervals for both the pre-intervention and post-intervention fitted trends.

The corresponding figure caption has also been updated accordingly.

## Comment 8

Data Availability Statement and Response Letter should be consistent.

### Response

Thank you for identifying this inconsistency.

The Data Availability Statement has been fully revised to comply with the current PLOS ONE data policy.

The manuscript now consistently states that:

- raw data originate from publicly accessible third-party sources (NBA Stats API and Basketball-Reference);
- the minimal derived datasets required to reproduce the analyses are provided as Supporting Information;
- all relevant derived data underlying the findings are available within the manuscript and its Supporting Information files.

The previous inconsistent wording referring to data being available upon request has been removed.

# Closing Statement

We are grateful to the Academic Editor and both reviewers for their careful evaluation and constructive recommendations. Their comments have substantially improved the quality, transparency, and statistical rigor of this work.

We hope that the revised manuscript now satisfactorily addresses all concerns and will be suitable for publication in **PLOS ONE**.

Thank you very much for your time and consideration.

Sincerely,

**Shuming Cui, Zhenxing Yang, Hemu Du, Rongheng Liu, Shijie Liu**
