# Supplementary material for: Shot-location polarization and post-2018 pace stabilization in the NBA: Evidence from 21 seasons of team and shot-coordinate data (2004–2025)
Source: PLoS One. 2026 Sep 18;21(9):e0358360. doi: 10.1371/journal.pone.0358360 (PMC13588525; doi:10.1371/journal.pone.0358360)
Supplement: Supplementary file 4 [file pone.0358360.s007.docx]

# Response to Reviewers

**Manuscript ID:** PONE-D-26-10048R2

**Title:** Shot-location polarization and post-2018 pace stabilization in the NBA: evidence from 21 seasons of team and shot-coordinate data (2004–2025)

Dear Academic Editor and Reviewer,

We sincerely thank the Academic Editor and Reviewer #2 for the careful evaluation of our revised manuscript. We greatly appreciate the reviewer’s detailed assessment and independent verification of the statistical analyses. The constructive comments have helped us further improve the methodological transparency, citation accuracy, statistical reporting, and presentation of the manuscript.

Following the reviewer’s suggestions, we have carefully revised the manuscript throughout. The principal revisions include:

- strengthening the Introduction with additional and more appropriate supporting references;
- improving citation accuracy by aligning references with the specific claims they support;
- clarifying the reproducibility and limitations of the NBA Stats shot-coordinate data;
- reporting marginal and conditional R² values for all linear mixed-effects models;
- adding a sensitivity analysis based on minimum detectable effects for the interrupted time-series analysis;
- adding 95% confidence intervals for all regression coefficients reported in Table 4;
- correcting the confidence interval presentation for TOV% in Table 1;
- strengthening the basketball-specific discussion using additional empirical literature;
- clarifying the interpretation of Figure 1; and
- further limiting causal language throughout the manuscript.

All revisions have been incorporated into the revised manuscript and are highlighted in the tracked-changes version.

Below we respond point by point.

# Academic Editor

### Comment

Dear authors, thank you for your interest in publishing in PLOS ONE. Please address the suggestions made by the reviewer.

### Response

We sincerely thank the Academic Editor for handling our manuscript. We have carefully considered and addressed all suggestions raised by Reviewer #2. Detailed responses are provided below.

# Reviewer #2

We sincerely thank Reviewer #2 for the exceptionally careful evaluation of our manuscript. We greatly appreciate the reviewer’s recognition that the major statistical concerns raised during the previous review rounds have been satisfactorily resolved. We are also grateful for the additional suggestions regarding citation support, methodological transparency, model reporting, and presentation quality. These comments have further strengthened the manuscript.

## Comment 1. Introduction and citation density

### Response

We agree and have revised the Introduction accordingly.

Specifically,

- the general statement regarding the speed–accuracy trade-off is now supported by the classical work of Fitts (1954);
- the basketball-specific statement concerning exercise intensity and execution accuracy is now supported by Mokou et al. (2016);
- Franks et al. (2015) has been cited where offensive spacing and defensive spatial structure are discussed;
- Goldsberry's CourtVision work has been cited to support statements regarding spatial analytics and shot-location measurement;
- an NBA official source has been added to support the historical discussion of the 2004 hand-checking rule changes.

Several sentences were also rewritten to improve citation accuracy and avoid attaching references to claims they do not directly support.

## Comment 2. Reliability of shot-coordinate data

### Response

We agree with the reviewer that deterministic reproducibility and external measurement validity represent different concepts.

Accordingly, the Methods and Limitations sections have been revised to clarify that:

- shot coordinates originate from the publicly accessible NBA Stats API;
- the same deterministic classification procedure was applied consistently across all seasons;
- identical coordinate inputs always produce identical spatial classifications;
- however, no independent coordinate dataset with comparable coverage across the entire 21-season period was available for external validation.

Rather than reporting unsupported measurement validity, we explicitly identify independent validation against tracking systems or video-based annotation as an important direction for future research. This revision more accurately reflects the evidential status of the underlying spatial data.

## Comment 3. Statistical reporting

### Response

We thank the reviewer for independently verifying our statistical calculations.

To further improve reporting transparency, we have additionally:

- reported marginal and conditional R² values for all linear mixed-effects models;
- incorporated these values into Tables 1 and 3 together with the corresponding table notes;
- expanded the Methods to explain the interpretation of marginal and conditional R².

For the interrupted time-series analysis, we also added a sensitivity analysis based on minimum detectable effects using HAC standard errors and finite-sample t-based inference. Because the study analyzed the complete population of available NBA seasons rather than a prospectively sampled cohort, we believe this provides a more appropriate assessment than a conventional post-hoc power calculation.

## Comment 4. Citation accuracy in the Discussion

### Response

We agree.

Section 4.3 has been revised so that the interpretation concerning offensive spacing and defensive geometry is now consistently supported by Franks et al. (2015). The citation has been replaced with Franks et al. (2015), which directly supports the interpretation regarding offensive spacing and defensive geometry.

The surrounding language has also been revised to present spacing-related explanations as plausible mechanisms requiring further investigation rather than definitive causal explanations.

## Comment 5. Tables and figures

### Response

We appreciate these suggestions.

Accordingly,

- 95% confidence intervals have been added to Table 4;
- the incorrect sign in the TOV% confidence interval reported in Table 1 has been corrected;
- Figure 1 has been retained without confidence intervals because each point represents the observed league-wide seasonal average rather than a sampled estimate. To avoid ambiguity, this rationale has been explicitly explained in the revised figure legend.

These revisions improve consistency across the presentation of statistical results.

## Comment 6. Basketball-specific literature

### Response

We appreciate these recommendations.

The Discussion has been strengthened by incorporating additional basketball-specific literature where directly relevant.

Specifically,

- empirical evidence regarding contemporary basketball physical-performance characteristics has been added to support discussion of the "new basketball body";
- basketball-specific repeated-sprint studies have been incorporated to strengthen discussion of the physiological demands associated with sustained high-tempo play.

These additions provide stronger basketball-specific support for the interpretations presented in Section 4.2.

## Comment 7. Overall interpretation

### Response

Following the reviewer’s comments, we carefully reviewed the entire manuscript and further distinguished descriptive association from causal interpretation.

Throughout the manuscript,

- SAI is consistently described as a shot-allocation metric rather than an efficiency measure;
- mixed-effects and regression results are interpreted as associations;
- the interrupted time-series analysis is described as evidence of temporal alignment and structural change rather than definitive causal effects of the 2018–19 rule modification;
- regression coefficients are interpreted as conditional descriptive associations given the observed multicollinearity.

These revisions ensure that the interpretation remains fully consistent with the observational design of the study.

# Closing Statement

We sincerely thank the Academic Editor and Reviewer #2 for their careful evaluation and constructive suggestions. Their comments have substantially improved the clarity, methodological transparency, statistical reporting, and overall quality of this manuscript.

We believe that the revised manuscript has addressed all remaining comments raised during this review round. We hope that the manuscript is now suitable for publication in PLOS ONE.

Thank you very much for your time and consideration.

Sincerely,

**Shuming Cui, Zhenxing Yang, Hemu Du, Rongheng Liu, and Shijie Liu**
