# Supplementary material for: Shot-location polarization and post-2018 pace stabilization in the NBA: Evidence from 21 seasons of team and shot-coordinate data (2004–2025)
Source: PLoS One. 2026 Sep 18;21(9):e0358360. doi: 10.1371/journal.pone.0358360 (PMC13588525; doi:10.1371/journal.pone.0358360)
Supplement: Peer review history

# Peer review: Decision Letter 0

**DOI:** 10.1371/journal.pone.0358360.r001

**Authors:** Gustavo Costa

13 Apr 2026

Dear Dr. cui,

Thank you for submitting your manuscript to PLOS ONE. After careful consideration, we feel that it has merit but does not fully meet PLOS ONE’s publication criteria as it currently stands. Therefore, we invite you to submit a revised version of the manuscript that addresses the points raised during the review process.

==============================

**ACADEMIC EDITOR:**

Dear Authors,

Thank you for submitting your manuscript to PLOS ONE.

After editorial assessment and consideration of the reviewers’ comments, your manuscript requires major revisions before it can be considered further for publication. Both reviewers identified substantial issues that must be addressed.

Please revise the manuscript within the deadline stipulated by PLOS ONE. When preparing your resubmission, ensure that you:

1. address all reviewer comments carefully and comprehensively;
2. prepare a detailed point-by-point response letter for each reviewer, indicating how each comment was addressed;
3. retain clear tracked changes, highlighted revisions, or other visible markings in the manuscript so that all modifications can be readily identified during reassessment.

In your response letter, please indicate precisely where each change has been made in the revised manuscript. Where you do not agree with a reviewer’s suggestion, provide a clear and respectful scientific justification.

Once your revised files are ready, please upload them through the journal submission system within the assigned revision period.

We look forward to receiving your revised manuscript.

Sincerely,

Gustavo De Conti Teixeira Costa

==============================

Please submit your revised manuscript by May 28 2026 11:59PM. If you will need more time than this to complete your revisions, please reply to this message or contact the journal office at plosone@plos.org. When you're ready to submit your revision, log on to https://www.editorialmanager.com/pone/ and select the 'Submissions Needing Revision' folder to locate your manuscript file.

- A letter that responds to each point raised by the academic editor and reviewer(s). You should upload this letter as a separate file labeled 'Response to Reviewers'.
- A marked-up copy of your manuscript that highlights changes made to the original version. You should upload this as a separate file labeled 'Revised Manuscript with Track Changes'.
- An unmarked version of your revised paper without tracked changes. You should upload this as a separate file labeled 'Manuscript'.

If you would like to make changes to your financial disclosure, please include your updated statement in your cover letter. Guidelines for resubmitting your figure files are available below the reviewer comments at the end of this letter.

If applicable, we recommend that you deposit your laboratory protocols in protocols.io to enhance the reproducibility of your results. Protocols.io assigns your protocol its own identifier (DOI) so that it can be cited independently in the future. For instructions see: https://journals.plos.org/plosone/s/submission-guidelines#loc-laboratory-protocols. Additionally, PLOS ONE offers an option for publishing peer-reviewed Lab Protocol articles, which describe protocols hosted on protocols.io. Read more information on sharing protocols at https://plos.org/protocols?utm_medium=editorial-email&utm_source=authorletters&utm_campaign=protocols.

As the corresponding author, your ORCID iD is verified in the submission system and will appear in the published article. PLOS supports the use of ORCID, and we encourage all coauthors to register for an ORCID iD and use it as well. Please encourage your coauthors to verify their ORCID iD within the submission system before final acceptance, as unverified ORCID iDs will not appear in the published article. *Only*  the individual author can complete the verification step; PLOS staff *cannot*  verify ORCID iDs on behalf of authors.

We look forward to receiving your revised manuscript.

Kind regards,

Gustavo De Conti Teixeira Costa, Ph.D

Academic Editor

PLOS One

Journal Requirements:

When submitting your revision, we need you to address these additional requirements.

1. Please ensure that your manuscript meets PLOS ONE's style requirements, including those for file naming. The PLOS ONE style templates can be found at

https://journals.plos.org/plosone/s/file?id=wjVg/PLOSOne_formatting_sample_main_body.pdf and

https://journals.plos.org/plosone/s/file?id=ba62/PLOSOne_formatting_sample_title_authors_affiliations.pdf

2. n this instance it seems there may be acceptable restrictions in place that prevent the public sharing of your minimal data. However, in line with our goal of ensuring long-term data availability to all interested researchers, PLOS’ Data Policy states that authors cannot be the sole named individuals responsible for ensuring data access (http://journals.plos.org/plosone/s/data-availability#loc-acceptable-data-sharing-methods).

Data requests to a non-author institutional point of contact, such as a data access or ethics committee, helps guarantee long term stability and availability of data. Providing interested researchers with a durable point of contact ensures data will be accessible even if an author changes email addresses, institutions, or becomes unavailable to answer requests.

Before we proceed with your manuscript, please also provide non-author contact information (phone/email/hyperlink) for a data access committee, ethics committee, or other institutional body to which data requests may be sent. If no institutional body is available to respond to requests for your minimal data, please consider if there any institutional representatives who did not collaborate in the study, and are not listed as authors on the manuscript, who would be able to hold the data and respond to external requests for data access? If so, please provide their contact information (i.e., email address). Please also provide details on how you will ensure persistent or long-term data storage and availability.

3. If the reviewer comments include a recommendation to cite specific previously published works, please review and evaluate these publications to determine whether they are relevant and should be cited. There is no requirement to cite these works unless the editor has indicated otherwise.

[Note: HTML markup is below. Please do not edit.]

Reviewers' comments:

Reviewer's Responses to Questions

**Comments to the Author**

1. Is the manuscript technically sound, and do the data support the conclusions?

Reviewer #1: Yes

Reviewer #2: Yes

**********

2. Has the statistical analysis been performed appropriately and rigorously?

Reviewer #1: Yes

Reviewer #2: Yes

**********

3. Have the authors made all data underlying the findings in their manuscript fully available?

The PLOS Data policy

Reviewer #1: Yes

Reviewer #2: Yes

**********

4. Is the manuscript presented in an intelligible fashion and written in standard English?

Reviewer #1: Yes

Reviewer #2: Yes

**********

Reviewer #1: Journal: Plos One

Id article: PONE-D-26-10048

Article title: Shot-location polarization and post-2018 pace stabilization in the NBA: evidence from 21 seasons of team and shot-coordinate data (2004–2025).

General evaluation.

• Relevance of the topic: Excellent.

• Introduction: Good.

• Methodology: Regular. It has to be improved.

• Results: Good.

• Discussion: Good.

• Conclusions: Good.

• References: Bad. They are shown in various styles (APA and Vancouver)

My decision is “Major revision is required”, to be evaluated again after the author’s corrections.

General comments.

The manuscript addresses a relevant and current topic in professional basketball analysis: the evolution of the pace of play and the spatial distribution of shots in the NBA. The approach combines longitudinal data, spatial analysis, and statistical models, which is appropriate for the stated objectives. The article is clear, well-structured, and supported by pertinent literature.

However, the manuscript presents methodological limitations, causal interpretation issues, insufficient definitions, and needs greater rigor in the presentation of results, especially in the statistical section and in the validation of the SAI index. Improvements are also needed in academic writing, conceptual precision, and analytical transparency.

• Several sections need linguistic editing to improve fluency and accuracy.

• Review consistency in the use of terms: “rim,” “restricted area,” “paint.”

• Clarify whether the 2024–25 data are complete or partial.

• Include a diagram of the data flow (API → cleaning → analysis).

• Review equation formatting (some appear with conversion errors).

• Add more recent references on spatial analysis in basketball (2020–2024).

• The abstract mentions >420,000 shots; verify if this matches the final dataset.

• Review section numbering (there are jumps and duplications).

Also, it is necessary to add a section related to the strengths and practical applications derived from the study.

Specific comments.

1.1. Conceptual justification of the SAI (Spatial Allocation Index)

The SAI is an interesting idea, but its definition is overly simple and not sufficiently validated.

The manuscript states:

“SAI captures shot-location allocation rather than shooting efficiency…” “SAI = (FGARim + FGA3PT) / FGATotal × 100%”

Issues:

• The index does not incorporate the substantial heterogeneity within the three-point area (corners vs. above-the-break).

• It does not distinguish between contested and uncontested shots, even though the paper itself cites literature on EPV and spatial decision-making.

• It is not justified why the 4-foot threshold for “rim” is sufficient.

• It is not compared against existing metrics (e.g., shot-chart entropy, spatial Gini, location variance).

1.2. Lack of detail on NBA API data extraction and cleaning

The manuscript states:

“We queried the shotchartdetail endpoint… and collected Cartesian coordinates.”

However, it does not specify:

• How shots without coordinates were handled.

• How duplicates, errors, or inconsistencies were treated.

• Whether playoffs were included or only the regular season.

• How coordinates were standardized across seasons (API changes).

• Whether endpoint stability was checked over 21 years.

1.3. Temporal segmentation: the ITS model is underspecified

The segmented regression model is presented as follows:

“Pace = β0 + β1 t + β2 Dt + β3 (t – T*)Dt + ε”

Issues:

• No confidence intervals are reported—only slopes.

• No autocorrelation diagnostics are shown (Durbin–Watson, ACF/PACF).

• It is not justified why the breakpoint is fixed in 2018 instead of being estimated.

• It is not assessed whether there is prior non-linearity (e.g., splines).

• The possibility of structural confounders is not discussed (e.g., changes in spacing, coaching, roster construction).

1.4. Overly strong causal interpretation

Although the paper claims it does not make strong causal inferences, in several places it suggests causality:

“The 2018–19 reset aligned with a step change in tempo…”

“The rule change may have contributed…”

The design does not support causal inference, because:

• There is no control group.

• External shocks are not modeled (COVID seasons, rule-enforcement changes).

• Changes in roster construction are not controlled for (e.g., big-men spacing).

• Differential effects by team are not evaluated.

1.5. The “safe velocity” concept is interesting but insufficiently grounded

The manuscript states:

“We refer to this descriptive pattern as ‘safe velocity’…”

Issues:

• This is a new concept without empirical validation.

• It is not shown that the reduction in TOV% is a consequence of the increase in pace.

• The relationship is not analyzed at the possession or player level.

• Potential mechanisms are not evaluated (spacing, decision speed, role specialization).

1.6. Lack of visual and quantitative analysis of the heatmaps

The manuscript mentions:

“Figure 2 illustrates changes in shot-location density…”

But it does not include:

• Spatial divergence metrics (KL divergence, Earth Mover’s Distance).

• A quantitative comparison between maps.

• Validation that the observed changes are not due to noise or sampling variation.

Reviewer #2: This manuscript analyses 21 NBA seasons (2004–05 to 2024–25) to describe shot-location polarization and pace dynamics around the 2018–19 shot-clock reset, combining team-season metrics with shot-coordinate data from the NBA Stats API. The authors introduce a Spatial Allocation Index (SAI), apply segmented regression within an interrupted time-series framework, and fit a multivariate OLS on Offensive Rating. The topic is timely and the dataset is substantial.

Strengths

The study is internally coherent and the research question is well framed. The combination of macro-level team data with micro-level shot coordinates is a strength, and the 21-season horizon allows long-run trends to be distinguished from short-term fluctuations. The interrupted time-series design is appropriate for evaluating the 2018–19 rule change, and the authors are commendably cautious in framing findings as associations rather than causal effects. Figures 2 and 4 convey the main messages effectively, and the discussion of the “efficiency paradox” is conceptually interesting.

Weaknesses

Several methodological and reporting issues require attention. No a priori power analysis or sample-size justification is provided; although the dataset is large, a formal rationale for the unit of analysis (team-season vs. league-season) should be stated. Test-retest reliability of the shot-coordinate extraction pipeline (nba_api endpoints) is not reported, nor is there any validation reference demonstrating the scientific accuracy of these coordinates or the sampling frequency of the underlying optical tracking. Impossible p-values (“p < 0.001”) appear repeatedly without exact values; confidence intervals are missing for most estimates, including the segmented regression coefficients. The ITS model relies on a single breakpoint fixed a priori at 2018–19 without sensitivity analyses or HAC/Newey–West standard errors actually being reported (only mentioned as possible). The OLS on ORtg omits multicollinearity diagnostics (VIF), and residual autocorrelation is not tested despite the time-ordered structure. No correction for multiple comparisons is applied. Figure 1 shows error-free trend lines without error bars, and Table 1 reports means ± SD but the group comparison is treated as a cross-sectional t-test, ignoring temporal dependence. The manuscript contains internal inconsistencies (e.g., “decreased from from” on line 215) and the English would benefit from careful revision.

Statistics and reporting

Page 8, lines 214–216: replace “p < 0.001” with exact p-values and 95% CIs; correct the duplicated “from from”. Decimal separators are consistently periods, which is appropriate; however, several estimates (e.g., SAI = 58.98%, 69.99%) should be accompanied by uncertainty bounds. Page 13, lines 297–307: segmented regression coefficients (β1 = 0.443; β2 = 4.268; β3 = −0.700) must include SEs, 95% CIs and exact p-values, and HAC standard errors should be computed and reported, not merely suggested. Page 13, Table 3: report VIFs, adjusted R², Durbin–Watson, and exact p-values to at least three decimals. The t-test in Table 1 treats 300 and 330 team-seasons as independent observations, which overstates degrees of freedom given within-team and within-season clustering; a mixed-effects or clustered-SE approach would be more defensible.

Sample size and power

No a priori sample-size calculation is reported. Although n = 630 team-seasons is large, a post-hoc justification based on the minimum detectable effect for the segmented regression (particularly for β3, the post-intervention slope change) should be provided, together with a discussion of statistical power given only seven post-intervention seasons.

Device, instrument and reliability information

Page 10, lines 119–125: the manuscript must specify the sampling frequency of the NBA optical tracking system underlying the shot coordinates, the system manufacturer and model (e.g., the tracking provider), country of origin, software version of the nba_api package used for extraction, and a peer-reviewed reference validating the accuracy of the coordinates retrieved through this endpoint. Test-retest reliability should be demonstrated by re-extracting a random subset of seasons and reporting agreement statistics (ICC or Cohen’s κ on binned zones).

Figures and tables

Figure 1 (page 9) should include shaded confidence bands or error bars around both series; currently, the Pearson r is reported without acknowledgement that both series are strongly trended, inflating the correlation. Figure 4 (page 13) should display confidence bands around the pre- and post-intervention fitted lines and a counterfactual extrapolation of the pre-2018 trend. Table 1 claims mean ± SD but should be accompanied by within-period visual representation (boxplots) to convey dispersion. Table 3 should report standardized coefficients alongside unstandardized B values to aid interpretation.

Specific suggested improvements with references

Page 8, lines 32–36 (Introduction, section 1.1): the discussion of optical tracking and expected possession value would benefit from a broader methodological anchor on how performance indicators are selected and validated in team sports. I suggest integrating reference [A] here to strengthen the epistemological framing.

Page 9, lines 57–62 (section 1.2, “Efficiency Paradox”): the speed–accuracy framing is underdeveloped. Reference [B] provides a rigorous basis for linking neuromuscular and decision-making demands to performance under time pressure, and should be cited when introducing the paradox.

Page 9, lines 81–83 (section 1.3): the citation of Edwards et al. (2018) on metabolic demand is appropriate but isolated. Adding reference [C] would substantiate the physiological cost of repeated high-intensity efforts and justify the discussion of practical constraints on pace.

Page 11, lines 147–152 (Rationale for metric selection): the selection of Four Factors indicators is justified only through Cecchin (2022). Reference [D], which addresses the methodological rigour of performance indicator selection in invasion sports, should be added here to reinforce the rationale.

Page 12, lines 183–196 (section 2.3.2, Segmented Regression): the ITS framework is under-referenced. Reference [E] provides methodological guidance on interrupted time-series design and should be cited to support the modelling choices, particularly the handling of autocorrelation.

Page 20, lines 386–390 (Discussion, section 4.2): the claim about the “new basketball body” and agility demands should be supported by reference [F], which addresses the physical profile of elite basketball players and the implications for high-tempo play.

Page 22, lines 407–415 (section 4.3, Defensive Spacing): the discussion of defensive geometry would be strengthened by reference [G], which provides complementary evidence on how spatial constraints modulate performance outcomes in team sports.

Language quality

The English is generally comprehensible but contains awkward phrasing and at least one typographical error (“from from”, line 215). A thorough revision by a native English-speaking editor is recommended, with particular attention to the Discussion section where sentence structures become long and occasionally ambiguous.

Recommendation

Major Revision. The study addresses an interesting question with a substantial dataset, but methodological rigour in statistical reporting, reliability documentation, and figure presentation must be improved before the manuscript can be considered for publication.

Suggested references (full details)

[A] Impellizzeri FM, Marcora SM. Test validation in sport physiology: lessons learned from clinimetrics. International Journal of Sports Physiology and Performance. 2009;4(2):269–277.

[B] Ferioli D, Rampinini E, Bosio A, La Torre A, Azzolini M, Coutts AJ. The physical profile of adult male basketball players: Differences between competitive levels and playing positions. Journal of Sports Sciences. 2018;36(22):2567–2574.

[C] Stojanović E, Stojiljković N, Scanlan AT, Dalbo VJ, Berkelmans DM, Milanović Z. The activity demands and physiological responses encountered during basketball match-play: A systematic review. Sports Medicine. 2018;48(1):111–135.

[D] Hughes MD, Bartlett RM. The use of performance indicators in performance analysis. Journal of Sports Sciences. 2002;20(10):739–754.

[E] Bernal JL, Cummins S, Gasparrini A. Interrupted time series regression for the evaluation of public health interventions: a tutorial. International Journal of Epidemiology. 2017;46(1):348–355.

[F] Scanlan AT, Dascombe BJ, Reaburn P. A comparison of the activity demands of elite and sub-elite Australian men's basketball competition. Journal of Sports Sciences. 2011;29(11):1153–1160.

[G] Sampaio J, McGarry T, Calleja-González J, Jiménez Sáiz S, Schelling i del Alcázar X, Balciunas M. Exploring game performance in the National Basketball Association using player tracking data. PLoS ONE. 2015;10(7):e0132894.

**********

what does this mean?). If published, this will include your full peer review and any attached files.

If you choose “no”, your identity will remain anonymous but your review may still be made public.

**Do you want your identity to be public for this peer review?**  For information about this choice, including consent withdrawal, please see our Privacy Policy

Reviewer #1: **Yes:** Víctor Hernández-Beltrán

Reviewer #2: No

**********

[NOTE: If reviewer comments were submitted as an attachment file, they will be attached to this email and accessible via the submission site. Please log into your account, locate the manuscript record, and check for the action link "View Attachments". If this link does not appear, there are no attachment files.]

To ensure your figures meet our technical requirements, please review our figure guidelines: https://journals.plos.org/plosone/s/figures

You may also use PLOS’s free figure tool, NAAS, to help you prepare publication quality figures: https://journals.plos.org/plosone/s/figures#loc-tools-for-figure-preparation.

NAAS will assess whether your figures meet our technical requirements by comparing each figure against our figure specifications.

# Author comment: Author response to Decision Letter 1

**DOI:** 10.1371/journal.pone.0358360.r002

25 Jun 2026

Response to Reviewers

Manuscript ID: PONE-D-26-10048

Title: Shot-location polarization and post-2018 pace stabilization in the NBA: evidence from 21 seasons of team and shot-coordinate data (2004–2025)

Dear Academic Editor and Reviewers,

We sincerely thank the Academic Editor and the reviewers for their careful evaluation of our manuscript and for their constructive and insightful comments. We have revised the manuscript extensively in response to all suggestions. The revisions have improved methodological transparency, statistical reporting, theoretical justification, and overall clarity.

All revisions are marked using tracked changes in the revised manuscript. Below, we provide a detailed point-by-point response to each comment, including the locations of the main changes.

============================================================

EDITOR COMMENTS

============================================================

Comment:

Please clarify long-term data availability and ensure that the data underlying the findings are accessible.

Response:

Thank you for this important recommendation. We revised the Data Availability Statement to clarify the original data sources, data-processing procedures, and the availability of the derived datasets used in the study. The original shot-location data were retrieved from publicly accessible NBA Stats web endpoints through the open-source nba_api Python package, and team-season performance data were obtained from Basketball-Reference. To comply with the PLOS Data Policy, we have uploaded the minimal derived datasets necessary to reproduce the analyses, tables, and figures as Supporting Information files. These files include the derived team-season and league-season variables, variable definitions, and data-processing details used in the present study. The Data Availability Statement has also been revised to remove the previous “available upon request” wording.

Changes in manuscript:

Data Availability Statement revised (Page 22). Supporting Information files containing the derived datasets and variable information have been uploaded with the revised submission.

============================================================

REVIEWER #1

============================================================

Comment 1:

The conceptual justification and validation of the Spatial Allocation Index (SAI) require improvement.

Response:

We agree. We expanded the conceptual justification to clarify that SAI is a descriptive measure of shot-allocation structure rather than a direct measure of offensive effectiveness. To evaluate its practical relevance, we added validation analyses examining correlations between SAI and Offensive Rating (ORtg), True Shooting Percentage (TS%), effective Field Goal Percentage (eFG%), and Winning Percentage (Win%). The results support the construct validity of SAI as an indicator of modern shot-allocation patterns.

Changes in manuscript:

Section 2.3.1 expanded; new validation subsection and Table 3 added (Section 3.3; Page 12).

------------------------------------------------------------

Comment 2:

Provide additional details regarding NBA API extraction and preprocessing procedures.

Response:

We added methodological detail describing data acquisition, inclusion criteria, preprocessing workflow, and spatial classification procedures to improve reproducibility and transparency. Specifically, we clarified that the analysis was restricted to NBA regular-season data, described the use of the shotchartdetail endpoint, explained the handling of missing or invalid coordinate records, and described the procedures used to classify shot locations and construct derived variables.

Changes in manuscript:

Section 2.1 Data Acquisition and Preprocessing revised (Methods section).

------------------------------------------------------------

Comment 3:

The interrupted time-series model lacks confidence intervals, autocorrelation diagnostics, and justification for the selected breakpoint.

Response:

We substantially expanded the interrupted time-series (ITS) analysis and reporting. We now report standard errors, 95% confidence intervals, exact p-values, model fit indices, and Durbin–Watson statistics. HAC/Newey–West standard errors are used for inference. We also included a sensitivity analysis using alternative placebo breakpoints in adjacent seasons to evaluate robustness. The 2018–19 breakpoint was retained because it corresponds to the implementation of the offensive-rebound shot-clock reset and because the primary model showed the strongest and most interpretable structural signal.

Changes in manuscript:

Sections 2.3.2 and 3.5 revised; Figure 4 updated (Page 15).

------------------------------------------------------------

Comment 4:

Causal interpretations should be softened.

Response:

We agree and revised the manuscript throughout to emphasize temporal alignment and association rather than causal inference. We explicitly noted the observational nature of the design, the absence of a control group, and the possibility of concurrent structural changes in league strategy, roster construction, spacing, and rule enforcement. Terms implying direct causality were replaced with more appropriate language such as “associated with,” “aligned with,” and “temporally coincided with.”

Changes in manuscript:

Revisions throughout Abstract, Results, and Discussion; Figure 4 note updated (Page 15); Limitations expanded (Discussion).

------------------------------------------------------------

Comment 5:

The concept of “safe velocity” requires stronger justification.

Response:

We clarified that “safe velocity” is used as a descriptive interpretation of observed league-level patterns rather than a formally validated theoretical construct. We also revised the discussion to make clear that the present analysis cannot establish that changes in turnover rate were caused by changes in pace. Alternative explanations, including spacing, decision speed, role specialization, and roster construction, are now discussed more cautiously.

Changes in manuscript:

Section 3.1 and Discussion revised.

------------------------------------------------------------

Comment 6:

Heatmaps require stronger quantitative interpretation.

Response:

We clarified that the heatmaps serve primarily as descriptive visualizations of changes in shot-location density. Their interpretation is now supported by quantitative analyses presented in Table 2 and by the SAI results. We also note that future studies could quantify spatial divergence using metrics such as KL divergence or Earth Mover’s Distance, which are beyond the scope of the present descriptive analysis.

Changes in manuscript:

Section 3.2 revised; Table 2 and SAI results support the quantitative interpretation.

------------------------------------------------------------

Comment 7:

References use mixed citation styles.

Response:

All references were reformatted according to PLOS ONE/Vancouver-style reference requirements. In-text citations and the reference list were checked for consistency.

Changes in manuscript:

Reference list fully revised.

------------------------------------------------------------

Comment 8:

Add a discussion of strengths and practical applications.

Response:

A dedicated subsection discussing study strengths and practical implications for coaches, analysts, and player-development practitioners has been added. This section highlights the value of long-term league-level monitoring, shot-location analysis, and the practical implications of perimeter-oriented offensive structures.

Changes in manuscript:

New Section 4.5 (Strengths and Practical Applications) added (Discussion).

============================================================

REVIEWER #2

============================================================

Comment 1:

Provide stronger justification for the analytical design and league-level sample.

Response:

We clarified the rationale for using complete-population NBA league data and discussed the strengths and limitations of league-level analyses. We explained that the study aims to describe long-term structural changes in league-wide shot allocation and pace rather than estimate player- or possession-level causal mechanisms. We also clarified the analytical distinction between team-season observations and league-season time-series analyses.

Changes in manuscript:

Methods and Limitations revised.

------------------------------------------------------------

Comment 2:

Provide confidence intervals, exact p-values, and additional statistical diagnostics.

Response:

We added confidence intervals and diagnostics where appropriate, including adjusted R² values and Durbin–Watson statistics, and clarified interpretive limits due to the league-level time-series structure. For large-sample correlation analyses, very small p-values are reported using conventional threshold notation where appropriate, whereas segmented regression coefficients and regression model estimates include more detailed inferential information.

Changes in manuscript:

Table 3 (Page 12), Table 4 (Page 15), and Section 3.5 (Page 15) revised.

------------------------------------------------------------

Comment 3:

The interrupted time-series model should report HAC/Newey–West standard errors and sensitivity analyses.

Response:

HAC/Newey–West standard errors are now reported. We also included placebo-breakpoint sensitivity analyses in adjacent seasons to evaluate the robustness of breakpoint selection. These analyses support the use of 2018–19 as the primary breakpoint because it is both theoretically meaningful and empirically consistent with the observed change in league-average pace.

Changes in manuscript:

Sections 2.3.2 and 3.5 revised; Figure 4 updated (Page 15).

------------------------------------------------------------

Comment 4:

Figures should include confidence intervals or uncertainty estimates.

Response:

Figures were revised to include confidence information where appropriate. Figure 4 includes a 95% confidence band around the pre-intervention trend and an explanatory note regarding interpretation. We also revised figure captions and related text to avoid overstating causal conclusions.

Changes in manuscript:

Figure 4 updated (Page 15); figure captions revised where appropriate.

------------------------------------------------------------

Comment 5:

Table 3 and Table 4 require more complete statistical reporting.

Response:

Table 3 was expanded and supplemented with explanatory notes. Table 4 reports model-level statistics including R², adjusted R², F-statistics, and Durbin–Watson diagnostics. Standardized coefficients were also reported where appropriate to aid interpretation.

Changes in manuscript:

Table 3 (Page 12) and Table 4 (Page 15) revised.

------------------------------------------------------------

Comment 6:

Several recommended references should be considered.

Response:

The suggested references were reviewed and incorporated where relevant in the Introduction, Methods, and Discussion. These additions strengthened the framing of performance indicator selection, interrupted time-series analysis, basketball activity demands, and spatial/team-performance analysis.

Changes in manuscript:

References and in-text citations updated accordingly.

------------------------------------------------------------

Comment 7:

English language and typographical issues require correction.

Response:

The manuscript was proofread and edited to improve clarity, consistency, and readability. Identified typographical and formatting errors, including duplicated wording and inconsistent terminology, were corrected. Terminology related to “rim,” “restricted area,” and shot-location zones was also checked for consistency.

Changes in manuscript:

Revisions made throughout the manuscript.

============================================================

ADMINISTRATIVE UPDATES

============================================================

In addition to the scientific revisions described above, the affiliation information for the first author (Shuming Cui) has been updated to reflect the current institutional affiliation due to academic enrollment/progression. No authors have been added, removed, or reordered during the revision, and the author byline remains unchanged from the initial submission. We also corrected the corresponding author information in the submission system to ensure consistency with the manuscript: the corresponding author is Zhenxing Yang. These administrative updates do not represent an authorship change and do not affect the study design, data collection procedures, analyses, results, interpretation, or conclusions.

The erroneous reference to Figure 5 has also been removed from the manuscript. Figure 5 is no longer part of the submission; therefore, no Figure 5 file or Figure 5 legend is included.

The Data Availability Statement was revised to remove the previous “available upon request” wording. The minimal derived datasets necessary to reproduce the analyses, tables, and figures have been uploaded as Supporting Information files.

============================================================

We sincerely thank the Academic Editor and Reviewers for their valuable comments and suggestions. We believe that the revised manuscript has been substantially strengthened and we respectfully submit it for further consideration.

Sincerely,

Shuming Cui and co-authors

Attachment Submitted filename: Response_to_Reviewers_PONE-D-26-10048.docx

# Peer review: Decision Letter 1

**DOI:** 10.1371/journal.pone.0358360.r003

**Authors:** Gustavo Costa

10 Jul 2026

Dear Dr. yang,

Thank you for submitting your manuscript to PLOS ONE. After careful consideration, we feel that it has merit but does not fully meet PLOS ONE’s publication criteria as it currently stands. Therefore, we invite you to submit a revised version of the manuscript that addresses the points raised during the review process.

Please submit your revised manuscript by Aug 24 2026 11:59PM. If you will need more time than this to complete your revisions, please reply to this message or contact the journal office at plosone@plos.org. When you're ready to submit your revision, log on to https://www.editorialmanager.com/pone/ and select the 'Submissions Needing Revision' folder to locate your manuscript file.

- A letter that responds to each point raised by the academic editor and reviewer(s). You should upload this letter as a separate file labeled 'Response to Reviewers'.
- A marked-up copy of your manuscript that highlights changes made to the original version. You should upload this as a separate file labeled 'Revised Manuscript with Track Changes'.
- An unmarked version of your revised paper without tracked changes. You should upload this as a separate file labeled 'Manuscript'.

If you would like to make changes to your financial disclosure, please include your updated statement in your cover letter. Guidelines for resubmitting your figure files are available below the reviewer comments at the end of this letter.

If applicable, we recommend that you deposit your laboratory protocols in protocols.io to enhance the reproducibility of your results. Protocols.io assigns your protocol its own identifier (DOI) so that it can be cited independently in the future. For instructions see: https://journals.plos.org/plosone/s/submission-guidelines#loc-laboratory-protocols. Additionally, PLOS ONE offers an option for publishing peer-reviewed Lab Protocol articles, which describe protocols hosted on protocols.io. Read more information on sharing protocols at https://plos.org/protocols?utm_medium=editorial-email&utm_source=authorletters&utm_campaign=protocols.

As the corresponding author, your ORCID iD is verified in the submission system and will appear in the published article. PLOS supports the use of ORCID, and we encourage all coauthors to register for an ORCID iD and use it as well. Please encourage your coauthors to verify their ORCID iD within the submission system before final acceptance, as unverified ORCID iDs will not appear in the published article. *Only*  the individual author can complete the verification step; PLOS staff *cannot*  verify ORCID iDs on behalf of authors.

We look forward to receiving your revised manuscript.

Kind regards,

Gustavo De Conti Teixeira Costa, Ph.D

Academic Editor

PLOS One

Journal Requirements:

If the reviewer comments include a recommendation to cite specific previously published works, please review and evaluate these publications to determine whether they are relevant and should be cited. There is no requirement to cite these works unless the editor has indicated otherwise.

[Note: HTML markup is below. Please do not edit.]

Reviewers' comments:

Reviewer's Responses to Questions

**Comments to the Author**

Reviewer #1: All comments have been addressed

Reviewer #2: All comments have been addressed

**********

2. Is the manuscript technically sound, and do the data support the conclusions?

Reviewer #1: Yes

Reviewer #2: Partly

**********

3. Has the statistical analysis been performed appropriately and rigorously?

Reviewer #1: Yes

Reviewer #2: Yes

**********

4. Have the authors made all data underlying the findings in their manuscript fully available?

The PLOS Data policy

Reviewer #1: Yes

Reviewer #2: Yes

**********

5. Is the manuscript presented in an intelligible fashion and written in standard English?

Reviewer #1: Yes

Reviewer #2: Yes

**********

Reviewer #1: Dear authors,

I would like to congratulate you and thank you for considering and making all the changes and improvements recommended. Actually, the article presents a good structure and a correct framework to substantiate the study's aim.

However, I would like to note that in some cases you are mixing reference styles (APA and between square brackets). In the same line, sometimes you can read in the text "Error! Reference source not found". Please, take a look at this and correct the issue.

Overall, I want to encourage you to continuos investigating to the topic and its relevance in the sports performance.

Reviewer #2: This revised submission (PONE-D-26-10048R1) reports a large-scale, twenty-one-season description of NBA shot-location and tempo evolution, built on team-season records from Basketball-Reference and shot-coordinate data drawn from the NBA Stats API. The authors have responded conscientiously to a first round of review, adding a validation subsection for their Spatial Allocation Index, expanding the interrupted-time-series analysis with HAC standard errors and placebo breakpoints, softening causal language throughout, and adding a strengths-and-applications subsection. These are genuine improvements and the manuscript reads as substantially more mature than a typical first submission. The overall design is coherent: separating shot-location composition (SAI) from tempo (Pace) and from possession-adjusted output (ORtg) is a sensible way to disentangle three related but distinct phenomena, and the combination of a segmented regression around the 2018-19 rule change with a broader twenty-one-year descriptive trend is an appropriate way to ask whether the rule change was a trigger or merely a marker of an ongoing process.

At the same time, a close reading of the statistics, several unresolved cross-reference errors, and a comparison between the manuscript, the response letter, and the tracked-changes file surface a set of concerns that a careful reviewer should not let pass. The most consequential is a mismatch between the regression equation specified in the Methods (Equation 3, four predictors) and the regression actually reported in Table 4 (three predictors, turnover percentage silently absent). A second concern is that several of the descriptive comparisons in Table 1 and the correlations in Table 3 treat team-season observations as statistically independent when they are repeated measures on the same thirty franchises, which is a textbook pseudoreplication problem that inflates apparent precision. A third is that at least four in-text citations have failed as broken Word cross-reference fields ("Error! Reference source not found."), and three further references appear in the bibliography without any corresponding in-text citation, which together suggest that the reference list was edited after the citation calls were last checked. Finally, comparing the response letter bundled inside the manuscript PDF with the separately submitted Response_to_Reviewers.docx reveals that the two documents answer the editor's data-availability comment differently, and the specific remedy described in the docx version, a named non-author contact at Capital Normal University, does not appear anywhere in the manuscript text supplied. None of these issues undermines the core empirical narrative, but each is fixable and each would materially improve the manuscript's technical credibility.

Detailed Evaluation

Introduction. The four subsections of the Introduction are reasonably well cited, but not uniformly so, and the principle that in an introduction almost every substantive claim should carry a source is worth taking seriously here because several load-bearing sentences currently stand alone. The opening sentence of Section 1.1, "The NBA has shifted from a slower, position-based half-court style to today's space-and-pace approach" (line 33), is presented as an established premise but has no citation attached to it; a source such as Wang and Zheng (2022), already reference [9] in the list, would sit naturally there rather than only later at line 103. More importantly, the entire opening of Section 1.2, "In sport research, the speed-accuracy trade-off is often used to explain performance: as actions become faster, error rates tend to rise. Applied to the NBA, this view would suggest that a higher pace should be linked to more turnovers..." (lines 60 to 63), is asserted without any citation at all, even though it is the theoretical premise the whole subsection is built to test. Ardigò, Kuvacic, Dello Iacono, Dascanio and Padulo (2018), on the effect of heart rate on basketball three-point shot accuracy, is a directly on-topic source for this exact claim: it demonstrates, within basketball itself and specifically for the perimeter shot that is central to this manuscript's SAI construct, that shot accuracy is sensitive to the physiological arousal that accompanies faster play, which grounds the efficiency-paradox premise in basketball-specific rather than only generic sport-science evidence. Attaching it at line 61 would close this gap.

Section 1.3 states the mechanics of the 2018-19 shot-clock reset (lines 79 to 80) as a bare factual claim; reference [20], the NBA's own press release, is in the bibliography for exactly this purpose but is never actually called in the text, so the claim is currently unsupported even though the source already exists in the list. This is a simple linking fix rather than a substantive gap.

A more mechanical problem recurs several times and deserves attention because it changes how the Introduction and Methods actually read to an editor. At line 103 in the Study Objectives paragraph, at line 159 in the Rationale for metric selection paragraph, at line 213 in the Segmented Regression Model paragraph, and again at line 289 in Section 3.2, the in-text citation has been replaced by the literal string "Error! Reference source not found.", sometimes duplicated, which is the visible symptom of a broken Word cross-reference field. Based on the surrounding argument and the numbering pattern of the surviving citations, these locations were almost certainly meant to point to reference [9] (Wang and Zheng, twice), reference [11] (Hughes and Bartlett), and reference [13] (Bernal, Cummins and Gasparrini) respectively; the last of these is particularly unfortunate because it is the interrupted-time-series methodology paper the authors themselves describe as underpinning their whole segmented-regression approach (line 212), yet the citation meant to anchor that claim is currently invisible to a reader. All of these fields need to be refreshed before the reference list and the in-text apparatus actually agree with each other.

The reference list itself contains a related asymmetry: references [20], [21] (Oliver, 2004) and [22] (Shortridge, Goldsberry and Adams, 2014) appear at the end of the bibliography but are not cited anywhere in the body text that could be located. Their position immediately after reference [19], together with the fact that reviewer 2's comment 6 in the response letter explicitly states that several recommended references were incorporated in this revision round, suggests these three were added to the list as part of that response but the corresponding in-text citation calls were never actually inserted. This is worth flagging directly to the authors, since an editor or a technical checker at the production stage is very likely to raise it as an uncited-reference error.

Methods. The Spatial Allocation Index is a reasonable and clearly motivated composition measure, and the new validation subsection is a genuine strength of the revision. One conceptual point is worth raising, gently, rather than as a fatal flaw: SAI is validated against effective field-goal percentage, true-shooting percentage and offensive rating, but eFG% is defined to give three-point makes one and a half times the weight of two-point makes, and TS% and ORtg are likewise mechanically sensitive to the same rim-versus-perimeter shot mix that SAI is built from. Part of the observed correlation between SAI and eFG% (r = 0.71) is therefore closer to a definitional relationship than to an independent empirical validation, and the manuscript would be more precise if it acknowledged that the strongest of the four validation correlations is partly a formula artifact rather than purely evidence of construct validity (Section 3.3, around line 306).

A second methods point concerns the data-generating instrument itself. The shot-coordinate records that underlie the heatmaps, Table 2 and the SAI time series come from the NBA Stats shotchartdetail endpoint (line 126), but the manuscript never states the sampling or digitization method behind those court coordinates, which vendor or system produced them, what spatial resolution or accuracy they carry, or whether the capture methodology was constant across all twenty-one seasons. Given that NBA tracking technology and data infrastructure changed materially over this window, this is exactly the kind of instrument information that should accompany a measurement used to build a primary outcome variable, together with a citation supporting its positional accuracy; at present neither is provided, and the manuscript would benefit from a short methodological note addressing it, ideally alongside a comment on the reliability, in the test-retest sense, of the shot-classification procedure that assigns raw coordinates to rim, mid-range and three-point zones.

A third, more concrete methods issue is the mismatch between Equation 3 and Table 4. Equation 3 specifies Offensive Rating as a function of four predictors, Pace, 3PAr, TOV% and ORB% (line 238), but Table 4 reports a model with only three predictors, 3PAr, ORB% and Pace, and an F-statistic of F(3,17) that is only internally consistent with three predictors and twenty-one seasons of data, not four. Recomputing R-squared directly from the reported F-statistic and degrees of freedom gives 3 times 34.51 divided by (3 times 34.51 plus 17), which equals 0.86 and reproduces the reported 0.859 essentially exactly, confirming that the model actually estimated used three predictors, so TOV% has been silently dropped somewhere between the equation and the table. Either the equation should be corrected to match the estimated model, or the four-predictor model should be reported with an explicit reason for excluding TOV%; collinearity with the other three predictors would be a plausible and defensible reason, but it needs to be stated rather than left implicit. Relatedly, the text asserts that model diagnostics included variance inflation factor analyses (line 243), but no VIF values are reported anywhere in the manuscript or the table notes; since this diagnostic is invoked as evidence against collinearity, the actual VIF values for the retained predictors should be shown.

Results and statistics. The "safe velocity" framing introduced in Section 3.1 (line 253) is a useful descriptive label, and Mokou, Nikolaidis, Padulo and Apostolidis (2016), on the acute effect of exercise intensity on free-throw accuracy in young basketball players, is a relevant citation to attach there: it provides basketball-specific evidence, alongside the manuscript's own descriptive contrast, that shooting accuracy does not automatically deteriorate with higher game intensity, which is precisely the empirical pattern the authors are describing at the league level. Independently recomputing the Table 1 comparisons from the reported means, standard deviations and group sizes reproduces the reported t-values closely (Pace t = -28.49 recomputed as -28.49; 3PAr, ORtg, TS% and TOV% all reproduce within rounding of the reported figures), which is a reassuring sign that the underlying numbers are internally consistent rather than transcribed incorrectly. The corresponding standardized effect sizes, which the manuscript does not report, are large by conventional benchmarks, with Cohen's d approximately 2.27 for Pace, 2.40 for 3PAr, 1.04 for ORtg, 1.16 for TS%, and 1.73 in absolute value for TOV%, so the practical magnitude of the old-era-versus-new-era shift is not in question. A different problem is worth flagging: the 300 and 330 team-season observations in Table 1, and the 620 team-seasons in Table 3, are repeated measures on the same thirty franchises across ten to eleven consecutive seasons each, not independent draws. A standard two-sample t-test or Pearson correlation assumes independence, and clustering by team will typically inflate apparent precision; even a modest intraclass correlation of 0.1 to 0.3, combined with roughly twenty observations per team, already reduces the effective sample size in Table 3 from 620 to somewhere between about 90 and 210. This matters most for the weakest of the four SAI validation correlations, SAI versus winning percentage (r = 0.079, p = 0.049 as reported and as independently reproduced here), which sits right at the conventional significance threshold and is precisely the coefficient most likely to lose significance once team-level clustering is properly modeled, for example with a mixed-effects model carrying team as a random intercept, or with cluster-robust standard errors. The authors already use HAC and Newey-West standard errors for the time-series analysis in Section 3.5, which shows they are alert to dependence structures in their data; the same care should be extended to the team-season comparisons in Sections 3.1 and 3.3.

The interrupted-time-series model itself is well specified and its headline numbers are internally coherent: the reported net post-2018 slope of -0.257 possessions per season reproduces exactly from beta-one plus beta-three, 0.443 minus 0.700, and Figure 4's visual break matches the reported coefficients. One statistical subtlety is that with only twenty-one seasonal observations and four estimated parameters, the residual degrees of freedom are 17, yet recomputing each coefficient divided by its HAC standard error and referring that ratio to a t-distribution with 17 degrees of freedom gives two-sided p-values several orders of magnitude larger than those reported. For beta-one this gives p approximately 1.5 times ten to the minus five against a t(17) reference, rather than the reported 2.68 times ten to the minus nine, and for beta-three it gives p approximately 4.5 times ten to the minus six rather than the reported 4.09 times ten to the minus eleven; the reported values instead match a large-sample normal reference distribution almost exactly.

Figure 4 also reports a ninety-five percent confidence band only around the pre-intervention trend, as its own note states. Reviewer 2's comment 4 specifically asked for uncertainty estimates on the figures, and the post-2018 segment, which is the more novel and more discussed part of the finding, the flattening and reversal of Pace growth, is currently shown without any visualized uncertainty at all. Extending the same band to the post-intervention segment would make the figure consistent with what was requested.

Discussion. Sections 4.1 through 4.4 build a coherent narrative connecting the spatial and temporal findings, and the interpretive caution the authors show elsewhere, framing the shot-allocation convergence as an empirical rather than a formal equilibrium, is well judged. Two of the interpretive claims could be strengthened with basketball-specific physiological evidence that is not currently cited. The discussion of temporal compression and practical constraints on Pace (Section 4.2) already leans on Edwards et al. (2018) for the general metabolic cost of repeated high-intensity actions in basketball; Zagatto, Ardigò, Barbieri, Milioni, Dello Iacono, Ferreira Camargo and Padulo (2017), on the metabolic demand of a repeated-sprint-ability test with varying numbers of changes of direction in basketball players, would sit naturally alongside it at line 457, since it quantifies exactly the kind of load that a shorter offensive-rebound reset imposes and gives the diminishing-returns argument a more concrete physiological anchor than the general fatigue literature alone. Similarly, the discussion of declining turnover rates and more stable decision environments under contemporary spacing (Section 4.3, around line 484) would benefit from Padulo, Nikolaidis, Cular, Dello Iacono, Vando, Galasso, Lo Storto and Ardigò (2018), on the effect of heart rate on jump-shot accuracy in adolescent basketball players, which offers direct evidence that shot-execution quality tracks the athlete's physiological state during play, complementing the purely tactical, spacing-based explanation currently offered for why speed and ball security appear to coexist in the modern league.

Compliance items. This is an original observational analysis of publicly available league and shot-location data, not a systematic review or meta-analysis, so PRISMA reporting and PROSPERO registration are not applicable and their absence is not a deficiency. Decimal formatting is correct throughout, with the period consistently used as the decimal separator and at least two decimal places reported for effect estimates, coefficients and correlations; this is a genuine strength and required no correction. No human participants, biological specimens or animals are involved, so the ethics statement of N/A is appropriate.

Verification against the response letter. Comparing the point-by-point response letter against the manuscript body, most of the claimed changes are genuinely present: the SAI validation subsection and Table 3 answer reviewer 1's comment 1, the expanded data-acquisition description in Section 2.1 answers reviewer 1's comment 2, the HAC standard errors, confidence intervals and placebo-breakpoint sensitivity analysis in Section 3.5 answer both reviewer 1's comment 3 and reviewer 2's comment 3, the softened causal language throughout the Abstract, Results and Discussion answers reviewer 1's comment 4, and the new Section 4.5 on strengths and practical applications answers reviewer 1's comment 8; all of these were located and confirmed as implemented. Two items, however, do not fully match what is claimed. First, reviewer 2's comment 6 states that recommended references were incorporated in the Introduction, Methods and Discussion; as discussed above, three of the newly added references have no in-text citation call, and three existing citation points show broken cross-reference fields, so the incorporation appears only partially complete at the level of the actual reference apparatus, whatever its completeness at the level of prose. Second, and more importantly, the two response documents supplied disagree with each other on the editor's data-availability comment. The response text embedded in the manuscript PDF answers a comment about clarifying long-term data availability by describing the removal of available-upon-request wording and the addition of Supporting Information files. The separately submitted Response_to_Reviewers.docx answers what is presented as the same editor comment, but phrased as also requiring non-author contact information for data access, with a response naming a specific institutional contact at Capital Normal University and an email address. That named contact does not appear anywhere in the Data Availability Statement of either the clean manuscript or the tracked-changes manuscript supplied here, and the tracked-changes copy of the Data Availability Statement still contains the sentence stating that code and retrieval instructions are available from the corresponding author upon request, which is precisely the phrasing the first response letter claims was removed. Given the discrepancy between the two response documents, and between both of them and the manuscript text actually reviewed here, the authors should reconcile which version of the Data Availability Statement, and which version of the response letter, is the current and final one before resubmission.

**********

what does this mean?). If published, this will include your full peer review and any attached files.

If you choose “no”, your identity will remain anonymous but your review may still be made public.

**Do you want your identity to be public for this peer review?**  For information about this choice, including consent withdrawal, please see our Privacy Policy

Reviewer #1: **Yes:** Víctor Hernández-Beltrán

Reviewer #2: No

**********

[NOTE: If reviewer comments were submitted as an attachment file, they will be attached to this email and accessible via the submission site. Please log into your account, locate the manuscript record, and check for the action link "View Attachments". If this link does not appear, there are no attachment files.]

To ensure your figures meet our technical requirements, please review our figure guidelines: https://journals.plos.org/plosone/s/figures

You may also use PLOS’s free figure tool, NAAS, to help you prepare publication quality figures: https://journals.plos.org/plosone/s/figures#loc-tools-for-figure-preparation.

NAAS will assess whether your figures meet our technical requirements by comparing each figure against our figure specifications.

# Author comment: Author response to Decision Letter 2

**DOI:** 10.1371/journal.pone.0358360.r004

27 Jul 2026

Response to Reviewers

Manuscript ID: PONE-D-26-10048R1

Title: Shot-location polarization and post-2018 pace stabilization in the NBA: evidence from 21 seasons of team and shot-coordinate data (2004–2025)

Dear Academic Editor and Reviewers,

We sincerely thank the Academic Editor and both reviewers for the careful evaluation of our manuscript and for the constructive suggestions provided during the previous review round. The comments were extremely helpful in improving the statistical rigor, methodological transparency, and overall presentation of the manuscript.

In this revised version, we carefully addressed every comment. The principal revisions include:

�replacing analyses affected by repeated observations with linear mixed-effects models;

�revising the interrupted time-series (ITS) analysis using finite-sample t-based inference while retaining HAC/Newey–West standard errors;

�correcting the inconsistency between Equation 3 and Table 4;

�reporting variance inflation factors (VIFs);

�expanding the description of the shot-coordinate data source and classification procedure;

�clarifying the interpretation and limitations of the Spatial Allocation Index (SAI);

�correcting all broken cross-references and reference-style inconsistencies; and

�revising the Data Availability Statement to ensure consistency with the manuscript and PLOS ONE requirements.

All modifications have been incorporated into the revised manuscript and are highlighted in the tracked-changes version.

Below we provide a detailed point-by-point response.

Response to Reviewer #1

We sincerely thank Reviewer #1 for the positive evaluation of our revised manuscript and for the careful reading.

Comment 1

In some cases you are mixing reference styles (APA and between square brackets).

Response

Thank you for identifying this issue. We carefully reviewed the entire manuscript and standardized all citations to the journal's numbered reference format. All remaining APA-style citations have been removed to ensure consistency throughout the manuscript.

Comment 2

"Error! Reference source not found."

Response

Thank you for noting this formatting problem.

The manuscript was carefully checked and all broken Word cross-reference fields were corrected. We refreshed the document fields, verified all in-text citations against the reference list, and confirmed that no unresolved cross-reference errors remain in the revised manuscript.

We appreciate the reviewer for identifying this issue.

Response to Reviewer #2

We sincerely thank Reviewer #2 for the detailed and thoughtful evaluation. We greatly appreciate the recognition that the manuscript has substantially improved. The remaining comments have been carefully addressed as described below.

Comment 1

Table 1 and Table 3 treat repeated observations from NBA franchises as statistically independent.

Response

We fully agree.

To account for repeated observations from the same NBA franchises across seasons, all analyses previously based on independent-sample tests were replaced with linear mixed-effects models using franchise as a random intercept.

Specifically,

�Table 1 has been completely reanalyzed using linear mixed-effects models;

�Table 3 has likewise been reanalyzed using mixed-effects models;

�the corresponding Methods section has been expanded to describe the statistical model;

�all Results have been updated accordingly; and

�the Limitations section now explicitly explains how within-franchise dependence was addressed and acknowledges the remaining limitations of this modeling strategy.

Importantly, after accounting for repeated observations, the previously marginal association between SAI and winning percentage was no longer statistically significant (β = 0.00123, p = 0.076), whereas the remaining principal findings remained unchanged.

These revisions substantially improve the statistical validity of the manuscript.

Comment 2

Equation 3 specifies four predictors whereas Table 4 reports three predictors.

Response

Thank you for identifying this inconsistency.

The inconsistency resulted from an earlier model-development stage in which turnover percentage (TOV%) had been evaluated but was subsequently removed from the final regression model because of substantial multicollinearity and limited additional explanatory value.

Equation 3 has been revised to match the final model reported in Table 4. The regression model now consistently includes:

�Pace,

�3-Point Attempt Rate (3PAr), and

�Offensive Rebound Percentage (ORB%).

The Methods, equation, and regression results are now fully consistent.

Comment 3

Report the variance inflation factors (VIFs).

Response

Thank you for this suggestion.

Variance inflation factors have now been explicitly reported in both the Results section and the note accompanying Table 4.

The VIF values are:

�3PAr = 15.52

�ORB% = 5.71

�Pace = 24.86

The Results section further explains that these large values reflect strong shared temporal trends among league-level predictors and that regression coefficients should therefore be interpreted as conditional descriptive associations rather than independent causal effects.

Comment 4

Provide additional information regarding the shot-coordinate data source and reliability.

Response

Thank you for this valuable suggestion.

The Data Acquisition section has been expanded substantially.

Specifically, we now clarify that:

�shot coordinates were retrieved from the publicly accessible NBA Stats API via the ShotChartDetail endpoint;

�the NBA does not publicly document the underlying coordinate acquisition procedures or measurement uncertainty;

�identical spatial classification thresholds were applied consistently across all seasons; and

�because the classification procedure is deterministic, identical coordinates always produce identical spatial categories, although external validation against an independent coordinate source is not currently available.

These additions improve methodological transparency while accurately reflecting the publicly available information.

Comment 5

The validation of SAI partly reflects shared mathematical structure with eFG%, TS%, and ORtg.

Response

We agree with this observation.

The revised manuscript now explicitly acknowledges that the observed associations should be interpreted cautiously because eFG%, TS%, and ORtg are partly influenced by the same rim-versus-perimeter shot composition represented by SAI.

Accordingly, we now describe these analyses as evidence of practical association rather than independent construct validation.

Comment 6

Statistical inference for the interrupted time-series analysis appears to rely on large-sample normal inference despite only 21 observations.

Response

Thank you for this important statistical observation.

We agree that finite-sample inference is more appropriate for the interrupted time-series model.

Accordingly, while retaining HAC/Newey–West standard errors to account for serial correlation and heteroskedasticity, we revised the statistical inference to use a t distribution with 17 residual degrees of freedom.

Specifically,

�the Methods section now explicitly describes the finite-sample inference procedure;

�the Results section has been updated with revised confidence intervals and p-values;

�the interpretation of the interrupted time-series analysis remains unchanged.

The substantive conclusions are unaffected, but the statistical reporting is now more appropriate for the available sample size.

Comment 7

Post-intervention uncertainty should be shown in Figure 4.

Response

Thank you for this suggestion.

Figure 4 has been revised to display 95% confidence intervals for both the pre-intervention and post-intervention fitted trends.

The corresponding figure caption has also been updated accordingly.

Comment 8

Data Availability Statement and Response Letter should be consistent.

Response

Thank you for identifying this inconsistency.

The Data Availability Statement has been fully revised to comply with the current PLOS ONE data policy.

The manuscript now consistently states that:

�raw data originate from publicly accessible third-party sources (NBA Stats API and Basketball-Reference);

�the minimal derived datasets required to reproduce the analyses are provided as Supporting Information;

�all relevant derived data underlying the findings are available within the manuscript and its Supporting Information files.

The previous inconsistent wording referring to data being available upon request has been removed.

Closing Statement

We are grateful to the Academic Editor and both reviewers for their careful evaluation and constructive recommendations. Their comments have substantially improved the quality, transparency, and statistical rigor of this work.

We hope that the revised manuscript now satisfactorily addresses all concerns and will be suitable for publication in PLOS ONE.

Thank you very much for your time and consideration.

Sincerely,

Shuming Cui, Zhenxing Yang, Hemu Du, Rongheng Liu, Shijie Liu

Attachment Submitted filename: Response to Reviewers.docx

# Peer review: Decision Letter 2

**DOI:** 10.1371/journal.pone.0358360.r005

**Authors:** Gustavo Costa

4 Aug 2026

Dear Dr. yang,

Please submit your revised manuscript by Sep 18 2026 11:59PM. If you will need more time than this to complete your revisions, please reply to this message or contact the journal office at plosone@plos.org. When you're ready to submit your revision, log on to https://www.editorialmanager.com/pone/ and select the 'Submissions Needing Revision' folder to locate your manuscript file.

- A letter that responds to each point raised by the academic editor and reviewer(s). You should upload this letter as a separate file labeled 'Response to Reviewers'.
- A marked-up copy of your manuscript that highlights changes made to the original version. You should upload this as a separate file labeled 'Revised Manuscript with Track Changes'.
- An unmarked version of your revised paper without tracked changes. You should upload this as a separate file labeled 'Manuscript'.

If you would like to make changes to your financial disclosure, please include your updated statement in your cover letter. Guidelines for resubmitting your figure files are available below the reviewer comments at the end of this letter.

If applicable, we recommend that you deposit your laboratory protocols in protocols.io to enhance the reproducibility of your results. Protocols.io assigns your protocol its own identifier (DOI) so that it can be cited independently in the future. For instructions see: https://journals.plos.org/plosone/s/submission-guidelines#loc-laboratory-protocols. Additionally, PLOS ONE offers an option for publishing peer-reviewed Lab Protocol articles, which describe protocols hosted on protocols.io. Read more information on sharing protocols at https://plos.org/protocols?utm_medium=editorial-email&utm_source=authorletters&utm_campaign=protocols.

As the corresponding author, your ORCID iD is verified in the submission system and will appear in the published article. PLOS supports the use of ORCID, and we encourage all coauthors to register for an ORCID iD and use it as well. Please encourage your coauthors to verify their ORCID iD within the submission system before final acceptance, as unverified ORCID iDs will not appear in the published article. *Only*  the individual author can complete the verification step; PLOS staff *cannot*  verify ORCID iDs on behalf of authors.

We look forward to receiving your revised manuscript.

Kind regards,

Gustavo De Conti Teixeira Costa, Ph.D

Academic Editor

PLOS One

Journal Requirements:

1. If the reviewer comments include a recommendation to cite specific previously published works, please review and evaluate these publications to determine whether they are relevant and should be cited. There is no requirement to cite these works unless the editor has indicated otherwise.

Additional Editor Comments:

Dear authors, thank you for your interest in publishing in PLOS ONE. Please address the suggestions made by the reviewer. I look forward to receiving the revised version.

Best regards,

Gustavo De Conti

[Note: HTML markup is below. Please do not edit.]

Reviewer's Responses to Questions

**Comments to the Author**

Reviewer #2: All comments have been addressed

**********

2. Is the manuscript technically sound, and do the data support the conclusions?

Reviewer #2: Yes

**********

3. Has the statistical analysis been performed appropriately and rigorously?

Reviewer #2: Yes

**********

4. Have the authors made all data underlying the findings in their manuscript fully available?

The PLOS Data policy

Reviewer #2: Yes

**********

5. Is the manuscript presented in an intelligible fashion and written in standard English?

Reviewer #2: Yes

**********

Reviewer #2: The manuscript presents a well-organized quantitative study of NBA offensive evolution across 21 seasons, combining team-season indicators with shot-coordinate data to describe shot-location polarization and a tempo inflection around the 2018-19 shot-clock reset. The revision has already addressed the two previous review rounds thoroughly, replacing independent-sample comparisons with linear mixed-effects models that account for franchise clustering, correcting the Equation 3 and Table 4 mismatch, reporting variance inflation factors, and adding confidence bands to Figure 4. Independent recomputation of the reported statistics, detailed below, confirms that the coefficients, standard errors, confidence intervals, F-statistic, and residual degrees of freedom in Tables 1, 3 and 4 are internally consistent and reproducible from the values given in the text, which is a genuine strength rarely found at this level of completeness in secondary-data sports analytics papers. The main weaknesses lie not in the statistical machinery itself but in three areas: an introduction whose citation density is uneven, with several load-bearing claims left unreferenced or attached to sources that do not squarely support them; the absence of any independent check on the reliability of the deterministic shot-coordinate classification that underlies every downstream indicator; and a small number of presentation details in the tables and figures that would benefit from tightening before typesetting.

Introduction and citation density

Page 11, line 62, opens the Efficiency Paradox subsection with the claim that the speed-accuracy trade-off is used in sport research to explain performance, and that faster actions produce higher error rates. As written, this foundational statement carries no citation at all; the numbered reference that follows two lines later, at line 63 to 64, is attached instead to the applied NBA claim about turnovers, and reference [7] there is Ardigo, Kuvacic, Dello Iacono, Dascanio and Padulo's 2018 Frontiers in Physiology study on heart rate and basketball three-point shot accuracy, a paper about a physiological correlate of individual shooting precision rather than about the general speed-accuracy relationship or about team-level turnover behavior. The citation should be split: the foundational sentence needs a classical motor-control or sport-science source for the trade-off itself, and the applied basketball sentence would be better served by a study that actually measures shooting or ball-handling accuracy under increased physical or temporal demand. Mokou, Nikolaidis, Padulo and Apostolidis' 2016 Sport Science for Health paper on the acute effect of exercise intensity on free-throw accuracy in young basketball players is a closer match for this second sentence and would sit naturally at line 64.

A second gap appears at page 11, lines 71 to 73: "One possibility is that changes in spacing and offensive structure make some decisions more stable at higher tempo, but this mechanism requires more direct testing." This is a substantive causal hypothesis with no supporting citation, even though the manuscript already has the right source for it: Franks, Miller, Bornn and Goldsberry's 2015 Annals of Applied Statistics paper on the spatial structure of defensive strategies, cited later as reference [22] in section 4.3. Bringing that citation forward to this sentence would close the gap without adding a new source.

Page 12, lines 89 to 90, states that "earlier rule changes, such as the stricter enforcement of hand-checking in the mid-2000s, also influenced offensive freedom." This is a specific historical claim about NBA officiating policy and needs a citation of its own, most naturally an NBA rule-history source in the same style as reference [10]; none of the reference collections available for this review contain a suitable match, so the authors should source this directly.

Page 10, lines 39 to 40, closes the tracking-technology paragraph with "these tools also made spacing easier to measure, so teams began to focus more on where shots are taken, and less on fixed positional roles," again without a citation, although Goldsberry's CourtVision work, already reference [12] later in the manuscript, speaks directly to this claim and could be cited here as well as at its current location.

Data source reliability and measurement validation

The manuscript is candid, at lines 127 to 135 and again at lines 596 to 601, that the NBA Stats API does not document its underlying coordinate acquisition procedure and that the spatial classification, while deterministic, has not been checked against an independent coordinate source. This is the equivalent, for an archival data study, of the test-retest reliability that would be expected of a directly measured variable in an original article, and it should be treated with the same rigor. Asserting that a rule-based classifier reproduces the same category for the same input is a statement about internal consistency, not about accuracy relative to ground truth. A modest validation exercise, for instance cross-checking a random sample of shot coordinates from several seasons against publicly available play-by-play video or a second independent coordinate feed and reporting a simple agreement statistic, would convert this from an acknowledged limitation into a demonstrated property of the data and would strengthen every result built on the Spatial Allocation Index.

Statistical verification

Recomputation confirms the internal coherence of the reported figures. In Table 4, F(3,17) recomputes to 34.52 from R squared of 0.859 with three predictors and seventeen residual degrees of freedom, matching the reported 34.51, and the three individual t-values reproduce to within rounding from the listed coefficients and standard errors. The residual degrees of freedom of 17 are correct for 21 seasonal observations with four estimated parameters, both in the segmented regression and in the multivariate model, and the net post-2018 Pace slope recomputes to -0.257 from the reported pre-trend of 0.443 and slope change of -0.700, consistent with the "-0.26" cited in the abstract and conclusion. The 95 percent confidence intervals in Tables 1 and 3 reproduce closely from their standard errors under normal-approximation multipliers, which is expected given the underlying sample sizes of 630 franchise-seasons for Table 3 and 300 versus 330 for Table 1.

On sample size and power, the study is not built around a pre-specified subject sample but around the full population of 21 available NBA seasons, so a conventional a priori power calculation before data collection would not have been meaningful. What can and should be reported is a sensitivity analysis of the kind the manuscript's own methods emphasize elsewhere: given the HAC standard error of 0.074 on the pre-2018 slope and 17 residual degrees of freedom, the minimum effect detectable at 80 percent power and two-sided alpha of 0.05 is approximately 0.22 possessions per season, well below the observed 0.44, and the corresponding minimum detectable level change is approximately 2.11 possessions against an observed 4.27. Reporting these thresholds explicitly, rather than leaving the reader to infer adequacy from significant p-values alone, would pre-empt any suggestion that the twenty-one-season design is underpowered for its central claims, and is more defensible than a post-hoc observed-power statement computed from the achieved effect itself.

A related methodological point concerns Tables 1 and 3, both fitted as linear mixed-effects models with franchise as a random intercept. The manuscript reports fixed-effect coefficients, standard errors and p-values but does not report marginal or conditional R squared for these models, so the proportion of variance attributable to the fixed effect of era or of SAI, as distinct from between-franchise variance captured by the random intercept, is not directly visible to the reader. Adding these alongside the existing VIF diagnostics in Table 4 would give a fuller picture of model fit throughout the results and would parallel the multicollinearity caution already applied well to the OLS model. Nau, Naidoo and Nau's 2025 Muscles, Ligaments and Tendons Journal guide to interpreting statistics with confidence, written for exactly this kind of situation where correlated predictors complicate coefficient interpretation, would support the cautious "conditional descriptive association" language the authors already use around line 404 to 406 and could be cited there.

Citation accuracy

Beyond the introduction, one further instance of a citation attached to a claim it does not squarely support occurs at page 27, line 504 to 505, in section 4.3: "higher tempo may be sustainable precisely because spacing has lowered the frequency of high-pressure situations[23], allowing speed and ball security to coexist more readily." Reference [23] here is again the Padulo group's 2018 paper on heart rate and jump-shot accuracy in adolescent players, an individual physiological study that does not address team-level defensive spacing or passing-lane stability. Franks et al., already cited two sentences earlier in the same paragraph as reference [22] for exactly this spatial-defensive argument, is the reference that actually carries the claim, and consolidating the citation there rather than splitting it across [22] and [23] would remove the mismatch.

Tables, figures and presentation quality

The figures are generally strong. Figure 3 and Figure 4 both display shaded 95 percent confidence bands around the fitted trends, which is good practice and was added specifically in response to the previous review round for Figure 4. Figure 1, by contrast, plots the raw Offensive Rating and three-point rate time series with no uncertainty band at all, which is a minor inconsistency given that it anchors the paper's central "efficiency paradox" narrative; a shaded band or season-level error bar, even if only illustrative given these are league averages rather than sampled means, would bring it in line with the other three figures. Table 4 reports standard errors but not 95 percent confidence intervals for the individual regression coefficients, unlike Tables 1 and 3, and adding that column would make the four results tables consistent with one another. In Table 1, the confidence interval reported for the TOV% adjusted difference appears in the manuscript as "1.06 to 0.77" rather than "-1.06 to -0.77"; given that the adjusted difference itself is correctly signed as -0.92, this looks like a sign or line-wrap artifact in the table cell rather than a computational error, but it is worth a proofing pass before typesetting so the printed interval does not read as spanning entirely positive values. The English throughout is fluent and appropriately technical for PLOS ONE; the main stylistic opportunity is in the introduction, where the repeated framing "research notes," "research points out," and "research suggests" across four consecutive paragraphs becomes formulaic, and varying this phrasing while adding the missing citations discussed above would improve both readability and referencing density at the same time.

Strengthening the basketball-specific literature base

Two further points in the Discussion would benefit from additional support drawn from directly basketball-relevant applied literature. Section 4.2, at lines 469 to 474, introduces the idea of a changing "new basketball body" citing only Tamayo's 2022 conceptual piece. Empirical benchmarking data on basketball players' physical profiles would substantiate this claim with measured values rather than conceptual argument alone: Jawabreh, Tounsi, Racil, Padulo, Migliaccio, Russo and Trabelsi's 2025 paper in Children on physical performance in young male basketball players assessed by maturity status, its companion 2024 Muscles, Ligaments and Tendons Journal paper on reference values of physical performance in young female basketball players, and Aouichaoui, Tounsi, Racil, Padulo, Martone and Trabelsi's 2024 Applied Sciences paper on percentile values of physical performance in Tunisian basketball players across a wide age range, together give the "new basketball body" argument an evidence base spanning both sexes and multiple populations rather than resting on a single narrative source.

At lines 476 to 478, the discussion of repeated high-intensity actions and metabolic demand under compressed decision windows currently rests on Edwards et al.'s general fatigue-monitoring review, reference [11], and Zagatto et al.'s repeated-sprint test paper, reference [21]. Two further basketball-specific repeated-sprint studies, Attene, Nikolaidis, Bragazzi, Dello Iacono, Pizzolato, Zagatto, Dal Pupo, Oggianu, Migliaccio, Mannucci Pacini and Padulo's 2016 Frontiers in Physiology paper on the chronic effects of multidirectional repeated-sprint training in young basketball players, and its companion Part 1 paper by Padulo, Bragazzi, Nikolaidis, Dello Iacono, Attene, Pizzolato, Dal Pupo, Zagatto, Oggianu and Migliaccio in the same volume, would anchor this specific physiological-constraint argument directly in basketball players rather than in the broader sport-science literature alone, and both would sit naturally alongside the existing citations at that point in the paragraph.

Recommendation

The manuscript is close to being fully sound. Addressing the introduction's uncited or mismatched sentences identified above, adding a brief validation check or explicit caveat on shot-coordinate classification accuracy, reporting marginal and conditional R squared for the mixed-effects models alongside the existing VIF diagnostics, adding confidence intervals to Table 4 and a proofing pass on the Table 1 TOV% interval, and weaving in the seven references suggested here at the specific locations indicated would meaningfully strengthen the paper's referencing rigor and internal completeness without requiring any change to its substantive conclusions, which the independent recomputation reported above supports as reproducible from the values already in the text.

Suggested references

The following seven references are suggested for integration at the locations indicated above. They are listed here in full detail, in the scattered order in which they arise across the manuscript rather than in priority sequence.

Mokou E, Nikolaidis P, Padulo J, Apostolidis N. The acute effect of exercise intensity on free throws in young basketball players. Sport Sci Health. 2016;12(2):227-232.

Nau AM, Naidoo N, Nau T. The orthopedic surgeon's guide to statistics: interpreting research with confidence. Muscles Ligaments Tendons J. 2025;15(2):121-129. doi:10.32098/mltj.02.2025.01

Jawabreh L, Tounsi M, Racil G, Padulo J, Migliaccio GM, Russo L, Trabelsi Y. Specific Physical Performances of Young Male Basketball Players in Palestine: An Assessment by Maturity Status. Children. 2025;12(1):64. doi:10.3390/children12010064

Jawabreh L, Tounsi M, Racil G, Frizziero A, Russo L, Migliaccio GM, Padulo J, Trabelsi Y. Reference Values of Physical Performances in Young Female Basketball Players in Palestine: Effect of Maturity Status. Muscles Ligaments Tendons J. 2024;14(3):458-469. doi:10.32098/mltj.03.2024.09

Aouichaoui C, Tounsi M, Racil G, Padulo J, Martone D, Trabelsi Y. Percentile Values of Specific Physical Performances in Tunisian Basketball Players Aged 9 to 21: Considering Maturity Status. Appl Sci. 2024;14(23):10882. doi:10.3390/app142310882

Attene G, Nikolaidis PT, Bragazzi NP, Dello Iacono A, Pizzolato F, Zagatto AM, Dal Pupo J, Oggianu M, Migliaccio GM, Mannucci Pacini E, Padulo J. Repeated sprint ability in young basketball players (Part 2): The chronic effects of multidirection and of one change of direction are comparable in terms of physiological and performance responses. Front Physiol. 2016;7:262. doi:10.3389/fphys.2016.00262

Padulo J, Bragazzi NL, Nikolaidis PT, Dello Iacono A, Attene G, Pizzolato F, Dal Pupo J, Zagatto AM, Oggianu M, Migliaccio GM. Repeated sprint ability in young basketball players: multi-direction vs. one-change of direction (Part 1). Front Physiol. 2016;7:133. doi:10.3389/fphys.2016.00133

**********

what does this mean?). If published, this will include your full peer review and any attached files.

If you choose “no”, your identity will remain anonymous but your review may still be made public.

**Do you want your identity to be public for this peer review?**  For information about this choice, including consent withdrawal, please see our Privacy Policy

Reviewer #2: No

**********

[NOTE: If reviewer comments were submitted as an attachment file, they will be attached to this email and accessible via the submission site. Please log into your account, locate the manuscript record, and check for the action link "View Attachments". If this link does not appear, there are no attachment files.]

To ensure your figures meet our technical requirements, please review our figure guidelines: https://journals.plos.org/plosone/s/figures

You may also use PLOS’s free figure tool, NAAS, to help you prepare publication quality figures: https://journals.plos.org/plosone/s/figures#loc-tools-for-figure-preparation.

NAAS will assess whether your figures meet our technical requirements by comparing each figure against our figure specifications.

# Author comment: Author response to Decision Letter 3

**DOI:** 10.1371/journal.pone.0358360.r006

12 Aug 2026

Dear Academic Editor and Reviewer,

Thank you very much for your careful evaluation and constructive comments. We have carefully addressed all comments raised by the reviewer. A detailed point-by-point response is provided in the uploaded file entitled "Response to Reviewers".

We sincerely appreciate your time and consideration.

Sincerely,

Shuming Cui, Zhenxing Yang, Hemu Du, Rongheng Liu, and Shijie Li

Attachment Submitted filename: Response_to_Reviewers_auresp_3.docx

# Peer review: Decision Letter 3

**DOI:** 10.1371/journal.pone.0358360.r007

**Authors:** Gustavo Costa

31 Aug 2026

Shot-location polarization and post-2018 pace stabilization in the NBA: evidence from 21 seasons of team and shot-coordinate data (2004–2025)

PONE-D-26-10048R3

Dear Dr. yang,

We’re pleased to inform you that your manuscript has been judged scientifically suitable for publication and will be formally accepted for publication once it meets all outstanding technical requirements.

Within one week, you’ll receive an e-mail detailing the required amendments. When these have been addressed, you’ll receive a formal acceptance letter and your manuscript will be scheduled for publication.

An invoice will be generated when your article is formally accepted. Please note, if your institution has a publishing partnership with PLOS and your article meets the relevant criteria, all or part of your publication costs will be covered. Please make sure your user information is up-to-date by logging into Editorial Manager at Editorial Manager® and clicking the ‘Update My Information' link at the top of the page. For questions related to billing, please contact billing support.

If your institution or institutions have a press office, please notify them about your upcoming paper to help maximize its impact. If they’ll be preparing press materials, please inform our press team as soon as possible -- no later than 48 hours after receiving the formal acceptance. Your manuscript will remain under strict press embargo until 2 pm Eastern Time on the date of publication. For more information, please contact onepress@plos.org.

Kind regards,

Gustavo De Conti Teixeira Costa, Ph.D

Academic Editor

PLOS One

Additional Editor Comments (optional):

Reviewers' comments:

Reviewer's Responses to Questions

**Comments to the Author**

Reviewer #2: All comments have been addressed

**********

2. Is the manuscript technically sound, and do the data support the conclusions?

Reviewer #2: Partly

**********

3. Has the statistical analysis been performed appropriately and rigorously?

Reviewer #2: Yes

**********

4. Have the authors made all data underlying the findings in their manuscript fully available?

The PLOS Data policy

Reviewer #2: Yes

**********

5. Is the manuscript presented in an intelligible fashion and written in standard English?

Reviewer #2: Yes

**********

Reviewer #2: The study's principal strength is methodological discipline. Across twenty-one seasons the authors separate a compositional measure of shot allocation from measures of shooting and scoring efficiency, apply an interrupted time-series design with HAC-consistent inference to isolate the 2018-19 breakpoint, and support every non-significant or borderline result with a genuine minimum-detectable-effect sensitivity analysis rather than post-hoc observed power, which is the technically correct choice and one still rare in applied sport science. Diagnostic reporting is also unusually complete: variance inflation factors are disclosed and interpreted honestly where multicollinearity is severe, marginal and conditional R² values accompany every mixed-effects model, and causal language is consistently restrained in favour of “temporal alignment” and “conditional association.” The residual weaknesses are narrow: a handful of undefined acronyms, an ambiguity in the reporting scale of three variables, one duplicated in-text citation that appears to have survived into the clean file after being corrected in the tracked-changes file, a labelling mismatch in the accompanying response letter, and an unacknowledged change in the underlying optical-tracking infrastructure that sits close to the studied breakpoint.

Audit of the revision against the response letter

The uploaded “Response to Reviewers” identifies itself as addressing manuscript PONE-D-26-10048R2, yet it is bundled with the R3 submission whose cover sheet carries the R3 number; the editorial office should confirm which review round this letter actually answers before it enters the permanent record. Substantively, however, the ten changes the authors claim were made are traceable in the tracked manuscript: additional and better-targeted citations appear throughout the Introduction (for instance around line 34 to 40, where Goldsberry's CourtVision work and Terner and Franks now support the optical-tracking claim that previously lacked a direct source), marginal and conditional R² values have been added to the notes of Tables 1 and 3, the minimum-detectable-effect sensitivity analysis has been inserted into section 2.3.2 immediately after the Durbin-Watson statistic, 95% confidence intervals now appear in Table 4, and the inverted confidence interval sign for TOV% in Table 1 has been corrected. One inconsistency did not fully propagate: in section 2.3.1, the sentence beginning “The motivation comes from spatial analytics work” cites reference 16 twice in the clean manuscript (“[16][16]”), while the same sentence in the tracked-changes file correctly reads “[1][16]”; the clean file submitted for typesetting should be resynchronised with the corrected version.

Every inferential figure checked against its reported point estimate and standard error reproduces to the precision shown. The minimum detectable effects in section 2.3.2 (0.22 possessions/season for the pre-2018 slope, 2.11 for the level change) match a two-sided alpha of 0.05 and 80% power on 17 residual degrees of freedom exactly. The adjusted R² values in both the segmented-regression model (0.924 from 0.935) and the ORtg regression (0.834 from 0.859, with F(3,17)=34.51) are internally consistent with the reported R² and degrees of freedom, as is the net post-2018 Pace slope of -0.257, which rounds correctly to the “-0.26” reported in the Abstract and Conclusion. The t-values, p-values and 95% confidence intervals in Table 4 recompute correctly from B and S.E. for every predictor; the ORB% p-value is reported as 0.01 while the three other p-values in the same table carry three decimals, a cosmetic inconsistency worth aligning to 0.010. The reported sample sizes are also internally coherent: 300 and 330 team-seasons for the two eras sum to 630, matching the 21 seasons by 30 franchises used in section 3.3.

Section 2.2 gives Pace and ORtg a clear unit (“per 48 minutes,” “per 100 possessions”), but it does not state whether 3PAr, TOV% and ORB% are expressed as proportions on a 0-to-1 scale or as percentages on a 0-to-100 scale. The regression equation in section 3.4 and the slope in Table 1 both treat 3PAr as a 0-to-1 proportion, while TOV% and ORB% are reported as whole percentage points in Table 1 (13.59, not 0.1359); a single explicit sentence stating the scale for each variable would remove any risk of a reader misapplying the coefficients in Table 4. Two acronyms are used without ever being expanded: eFG%, which first appears in section 3.3 and again in Table 3 and its note, and HAC in “HAC/Newey-West standard errors” in section 2.3.2; FGA in equation (1) is likewise never formally written out as “field-goal attempts” even though the quantity is used throughout. NBA itself, while universally recognised, is technically also unexpanded at first use.

There is no physical instrument in this study, but the NBA Stats coordinate feed functions as one, and the manuscript's own candour about it (section 2.1, and again in the Limitations) is appropriate: the authors correctly distinguish deterministic reproducibility of their rule-based classification from external validation of the underlying coordinates, and they do not overstate reliability they have not demonstrated, which is the right standard. What is missing is any mention that the NBA's optical-tracking provider changed from SportVU to Second Spectrum at the start of the 2017-18 season, one season before the studied 2018-19 breakpoint, and again from Second Spectrum to Hawk-Eye in 2023-24, inside the “stabilised” post-intervention window the authors describe in section 3.5. Neither transition need have affected shot-coordinate classification, but given that the manuscript already flags uncertainty about the acquisition pipeline, a sentence noting these transitions and their timing relative to the breakpoint would pre-empt an obvious question and would cost the authors nothing, since their own segmented-regression sensitivity check against placebo breakpoints already speaks to the robustness of the 2018-19 estimate.

Three additions would strengthen specific, identifiable passages without inflating the reference list. In section 2.3.2, immediately after the sentence noting that “an a priori power calculation was not applicable,” Hoenig and Heisey's classical critique of post-hoc power directly supports the authors' own methodological choice and would give a reader unfamiliar with the sensitivity-analysis literature the primary source for why observed power was correctly avoided. In the Limitations, where the authors state that “external validation against an independent coordinate source was not available,” a study establishing the validity and reliability of a standalone positional-tracking receiver against a reference standard offers a directly transferable precedent for how such external validation is normally conducted in sport science, even though the sport and tracking modality differ. Finally, in the same section 2.3.2 sensitivity-analysis paragraph, a paper establishing the minimal detectable change of a sport-specific test offers a conceptual parallel from reliability research to the effect-detection threshold the authors compute from a power standpoint; the two concepts are related but not interchangeable, and the text should keep that distinction explicit if the citation is added. No further sport-specific literature was identified that would add substantive value beyond what the manuscript already cites extensively.

Line 57 to 58 of the Introduction runs two sentences together after reference [9] (“mid-range attempts[9] Together, these trends”) with no terminal punctuation; a full stop is missing. The English throughout is otherwise fluent and precise, and the hedged, non-causal register the authors adopt in the Discussion reads naturally rather than defensively.

Minor Revision. Ask the authors to reconcile the response-letter manuscript ID, correct the duplicated citation in section 2.3.1 of the clean file, add one sentence defining the reporting scale of 3PAr, TOV% and ORB%, expand eFG%, HAC and FGA at first use, and add a brief note on the two tracking-provider transitions relative to the 2018-19 breakpoint. None of these items bears on the validity of the reported findings, which the independent statistical check above supports without qualification.

Suggested references (full detail)

Hoenig JM, Heisey DM. The Abuse of Power: The Pervasive Fallacy of Power Calculations for Data Analysis. Am Stat. 2001;55(1):19-24. doi:10.1198/000313001300339897.

Padulo J, Iuliano E, Brisola G, Dello Iacono A, Zagatto AM, Lupo C, Fuglsang T, Ardigò LP, Čular D. Validity and reliability of a standalone low-end 50-Hz GNSS receiver during running. Biol Sport. 2019;36(1):75-80. doi:10.5114/biolsport.2019.79974.

Čular D, Dhahbi W, Kolak I, Dello Iacono A, Bešlija T, Laffaye G, Padulo J. Reliability, Sensitivity, and Minimal Detectable Change of a New Specific Climbing Test for Assessing Asymmetry in Reach Technique. J Strength Cond Res. 2021;35(2):527-534. doi:10.1519/JSC.0000000000002694.

**********

what does this mean?). If published, this will include your full peer review and any attached files.

If you choose “no”, your identity will remain anonymous but your review may still be made public.

**Do you want your identity to be public for this peer review?**  For information about this choice, including consent withdrawal, please see our Privacy Policy

Reviewer #2: **Yes:** Johnny Padulo

**********

# Editor report: Acceptance letter

**DOI:** 10.1371/journal.pone.0358360.r008

**Authors:** Gustavo Costa

PONE-D-26-10048R3

PLOS One

Dear Dr. yang,

I'm pleased to inform you that your manuscript has been deemed suitable for publication in PLOS One. Congratulations! Your manuscript is now being handed over to our production team.

At this stage, our production department will prepare your paper for publication. This includes ensuring the following:

* All references, tables, and figures are properly cited

* All relevant supporting information is included in the manuscript submission,

* There are no issues that prevent the paper from being properly typeset

You will receive further instructions from the production team, including instructions on how to review your proof when it is ready. Please keep in mind that we are working through a large volume of accepted articles, so please give us a few days to review your paper and let you know the next and final steps.

Lastly, if your institution or institutions have a press office, please let them know about your upcoming paper now to help maximize its impact. If they'll be preparing press materials, please inform our press team within the next 48 hours. Your manuscript will remain under strict press embargo until 2 pm Eastern Time on the date of publication. For more information, please contact onepress@plos.org.

You will receive an invoice from PLOS for your publication fee after your manuscript has reached the completed accept phase. If you receive an email requesting payment before acceptance or for any other service, this may be a phishing scheme. Learn how to identify phishing emails and protect your accounts at https://explore.plos.org/phishing.

If we can help with anything else, please email us at customercare@plos.org.

Thank you for submitting your work to PLOS One and supporting open access.

Kind regards,

PLOS One Editorial Office Staff

on behalf of

Dr. Gustavo De Conti Teixeira Costa

Academic Editor

PLOS One
